# ERICH3: vesicular association and antidepressant treatment response

**DOI:** 10.1038/s41380-020-00940-y

**Published:** 2020-11-23

**Authors:** Duan Liu, Yongxian Zhuang, Lingxin Zhang, Huanyao Gao, Drew Neavin, Tania Carrillo-Roa, Yani Wang, Jia Yu, Sisi Qin, Daniel C. Kim, Erica Liu, Thanh Thanh Le Nguyen, Joanna M. Biernacka, Rima Kaddurah-Daouk, Boadie W. Dunlop, W. Edward Craighead, Helen S. Mayberg, Elisabeth B. Binder, Mark A. Frye, Liewei Wang, Richard M. Weinshilboum

**Affiliations:** 1 Division of Clinical Pharmacology, Department of Molecular Pharmacology and Experimental Therapeutics, Mayo Clinic, Rochester, MN USA; 2 Department of Translational Research in Psychiatry, Max Planck Institute of Psychiatry, Munich, Germany; 3 Xi’an No.1 Hospital, the First Affiliated Hospital of Northwest University, Xi’an, Shaanxi China; 4 Shaanxi Institute of Ophthalmology, Shaanxi Key Laboratory of Ophthalmology, Shaanxi Clinical Research Center for Ophthalmology Diseases, Xi’an, Shaanxi China; 5 Department of Psychiatry and Psychology, Mayo Clinic, Rochester, MN USA; 6 Department of Health Sciences Research, Mayo Clinic, Rochester, MN USA; 7 Department of Psychiatry and Behavioral Sciences, Duke University, Durham, NC USA; 8 Department of Medicine, Duke University, Durham, NC USA; 9 Duke Institute for Brain Sciences, Duke University, Durham, NC USA; 10 Department of Psychiatry and Behavioral Sciences, Emory University School of Medicine, Atlanta, GA USA; 11 Departments of Neurology and Neurosurgery, Icahn School of Medicine at Mount Sinai, New York, NY USA; 12 Present Address: Rubedo Life Sciences, Sunnyvale, CA USA; 13 Present Address: Centre for Cellular Genomics, Garvan Institute of Medical Research, Sydney, NSW Australia

**Keywords:** Genetics, Biomarkers, Molecular biology

## Abstract

Selective serotonin reuptake inhibitors (SSRIs) are standard of care for major depressive disorder (MDD) pharmacotherapy, but only approximately half of these patients remit on SSRI therapy. Our previous genome-wide association study identified a single-nucleotide polymorphism (SNP) signal across the glutamate-rich 3 (*ERICH3*) gene that was nearly genome-wide significantly associated with plasma serotonin (5-HT) concentrations, which were themselves associated with SSRI response for MDD patients enrolled in the Mayo Clinic PGRN-AMPS SSRI trial. In this study, we performed a meta-analysis which demonstrated that those SNPs were significantly associated with SSRI treatment outcomes in four independent MDD trials. However, the function of ERICH3 and molecular mechanism(s) by which it might be associated with plasma 5-HT concentrations and SSRI clinical response remained unclear. Therefore, we characterized the human *ERICH3* gene functionally and identified ERICH3 mRNA transcripts and protein isoforms that are highly expressed in central nervous system cells. Coimmunoprecipitation identified a series of ERICH3 interacting proteins including clathrin heavy chain which are known to play a role in vesicular function. Immunofluorescence showed ERICH3 colocalization with 5-HT in vesicle-like structures, and ERICH3 knock-out dramatically decreased 5-HT staining in SK-N-SH cells as well as 5-HT concentrations in the culture media and cell lysates without changing the expression of 5-HT synthesizing or metabolizing enzymes. Finally, immunofluorescence also showed ERICH3 colocalization with dopamine in human iPSC-derived neurons. These results suggest that ERICH3 may play a significant role in vesicular function in serotonergic and other neuronal cell types, which might help explain its association with antidepressant treatment response.

## Introduction

Major depressive disorder (MDD) is the most common psychiatric disease and a major cause of medical disability worldwide [1, 2]. Relative deficiency of the monoamine neurotransmitter serotonin (5-HT) appears to play a role in the pathophysiology of MDD and, as a result, drugs such as selective serotonin reuptake inhibitors (SSRIs) that enhance serotonergic neurotransmission are used to treat MDD. SSRIs are standard-of-care pharmacotherapy for MDD [3], but many MDD patients fail to respond to SSRI therapy and, even in those who do respond, the response may be delayed for weeks or months [4]. Several genome-wide association studies (GWAS) for SSRI treatment outcomes have been performed [5–9], but few of the top signals from those GWAS have been replicated or functionally validated [10, 11]. This situation may result, in part, from MDD phenotypic heterogeneity and/or the lack of biologically based phenotypes [12].

In an effort to move beyond genomics alone, we previously applied a “pharmacometabolomics-informed pharmacogenomic” research strategy [13–15] with the goal of discovering mechanisms related to variation in SSRI response [15]. That approach began with the association of plasma metabolite concentrations with SSRI response, followed by GWAS using concentrations of those metabolites as phenotypes to identify genetic loci associated with variation in metabolite concentrations—concentrations that represented biologically-based quantitative phenotypes. Of the metabolites that we assayed, plasma 5-HT concentrations at baseline as well as changes in plasma 5-HT concentrations after SSRI treatment were most significantly associated with SSRI response in the MDD patients whom we studied [15, 16]. The association of plasma 5-HT concentrations with SSRI response has now been replicated in two independent studies [17, 18].

GWAS for baseline plasma 5-HT concentrations in our MDD patients identified a single-nucleotide polymorphism (SNP) signal across the glutamate-rich 3 (*ERICH3*) gene (rs696692, *P* = 9.28E–08; *N* = 290), which did not reach the genome-wide significant threshold (*P* ≤ 5.0E–08). That same SNP signal was also observed in our GWAS for change in plasma 5-HT concentrations after SSRI treatment [15]. One of the *ERICH3* SNPs (rs10580409, *P* = 1.12E–07) which was in tight linkage disequilibrium with the rs696692 “top” SNP (D’ = −0.98, *r*^2^ = 0.96 in European subjects as reported by the 1000 Genomes Project [19]) was a nonsynonymous SNP (nsSNP) associated with accelerated proteasome-mediated ERICH3 protein degradation [15]. In addition, we observed that changing ERICH3 expression by siRNA knock-down or cDNA over-expression in central nervous system (CNS) derived cell lines resulted in significant changes of 5-HT concentrations in the cell culture media [15]. We also found that the *ERICH3* rs10580409 nsSNP which was identified in our GWAS for plasma 5-HT concentrations in MDD patients, was also directly associated with SSRI response in independent SSRI treatment response GWAS [15], including those for the Sequenced Treatment Alternatives to Relieve Depression (STAR*D) [4] and the International SSRI Pharmacogenomics Consortium (ISPC) [9].

When we initially identified *ERICH3* with our GWAS for plasma 5-HT concentrations in MDD patients, as described in the preceding paragraph, it was named “chromosome 1 open reading frame 173 (*C1orf173*)” based on its map location in the human genome, and nothing was known about the gene or the protein that it encoded functionally. It was subsequently renamed “E-rich 3” as a result of the high content of glutamate (E) in its encoded amino acid sequence. A total of six *ERICH* genes, from *ERICH1* to *ERICH6*, have been identified in the human genome. However, with the exception of their high content of E, the proteins encoded by these genes do not display similarity in their amino acid sequences or protein domains. As a result, they may not be related functionally. None of the ERICH proteins have been well characterized and their functions are presently unknown. RNA sequencing (RNA-seq) [20–22] and mass-spectrometry-based proteomic data [23, 24] for different human tissues have shown that ERICH3 is most highly expressed in a variety of regions of the human brain, including the nucleus accumbens (basal ganglia) and frontal cortex (https://www.gtexportal.org/home/gene/ERICH3). In addition to the *ERICH3* nsSNP that we found to be associated with plasma 5-HT concentrations and SSRI response in MDD patients [15], another *ERICH3* SNP, rs114396406, has recently been associated with several depression-related phenotypes including risk for severe depression (*P* = 9.362E-05; OR = 0.926; *N* = 54,567), in a large cohort GWAS reported by the Global Biobank Engine using the UK Biobank dataset [25] (https://gbe.stanford.edu/). Transcriptome data generated by the PsychENCODE project also showed that ERICH3 mRNA was elevated in postmortem human brain samples from subjects with schizophrenia (FDR = 0.01) [26]. Taken together, these observations suggest that ERICH3 might be related to risk for psychiatric disorders such as depression and schizophrenia.

It is against this background that the series of experiments described subsequently was performed. Specifically, we set out to (1) define the *ERICH3* gene structure, (2) identify CNS cells in which ERICH3 was expressed, (3) define ERICH3 mRNA species and the proteins which those species encoded, (4) apply coimmunopreciptiation (Co-IP) to identify proteins that interact with ERICH3, many of which proved to be vesicle-related proteins, (5) to define the effect of ERICH3 knock-out (KO) on monoamine neurotransmitter vesicular function and finally, (6) to perform a meta-analysis to test association of the *ERICH3* rs11580409 SNP that alters the amino acid sequence of the encoded protein with SSRI response for MDD patients enrolled in four independent MDD SSRI trials. The results of those experiments are described in subsequent paragraphs, providing a foundation for understanding the contribution of ERICH3 to monoamine neurotransmitter function and perhaps other neurotransmitter systems in order to understand the possible contribution of this gene to the pathophysiology of neuropsychiatric disease.

## Materials and methods

### ERICH3 transcriptional analysis at the single cell level

Single-cell RNA-seq data generated using surgically-removed human cerebral cortical samples from 12 subjects [27, 28] were downloaded and combined for analysis. Those sequenced CNS cells were “clustered” by mRNA expression levels of marker genes and were visualized using previously published methods [29, 30]. RNA raw counts were mapped to the human reference genome GRCh38 (hg38) to quantify relative *ERICH3* exon and exon junction expression. RNA counts were further normalized by exon length and RNA-seq cDNA library size. Please see the Supplementary Text for details.

### ERICH3 protein characterization

Western blot analysis was used to characterize ERICH3 protein isoforms. cDNA constructs for use in overexpressing ERICH3 protein isoforms were created and transfected into HEK (human embryonic kidney) 293 T cells from which protein lysates were used as positive controls to test ERICH3 antibodies. Lysates from human primary neurons were also used for ERICH3 protein identification. The reagents used, including antibodies, plasmids, and other reagents are listed in Supplementary Table S1.

### ERICH3-interacting protein identification

Co-Immunoprecipitation (Co-IP) using ERICH3 antibody (Ab-Ex 14) was performed with SK-N-SH (human brain neuroblastoma) cells. Non-specific rabbit IgG was used as a negative control. Co-IP protein samples were separated by SDS-PAGE and bands were cut out of the gels for mass spectrometric (MS) analysis. MS results were validated by Western blot and immunofluorescence (IF) assays. See the Supplementary Text for details.

### ERICH3 relationship to 5-HT concentrations

SK-N-SH cells were edited by CRISPR/cas9 using two guide RNAs (gRNAs) targeting the *ERICH3* gene. SK-N-SH cells were then incubated with 40 µM 5-hydroxytryptophan (5-HTP), a compound that can be metabolized by DOPA decarboxylase (DDC) to form 5-HT. Concentrations of 5-HT and its primary metabolite, 5-HIAA, were assayed in the cell media and lysates by HPLC-with electrochemical detection. 5-HT in cells was also stained and visualized by IF. Protein levels of 5-HT metabolizing enzymes were quantified by Western blot analysis. Human iPSC-derived neurons were infected by lentivirus packaged with shRNAs for ERICH3 KD. Cell culture media were then collected for the detection of catecholamines and catecholamine metabolites. See the Supplementary Text for details.

### Association of the ERICH3 rs11580409 SNP with antidepressant treatment response

The possible association between the *ERICH3* rs11580409 SNP genotype and antidepressant treatment responses in the STAR*D [4], ISPC [9], PReDICT [31, 32], and PGRN-AMPS [8, 33] studies was determined using logistic regression after adjusting for age, gender, and race. Results of those 4 studies were combined for an inverse-variance weighted fixed-effects meta-analysis using the “rmeta” package in R. Treatment “response” was defined as a reduction of at least 50% in depression score as determined by use of the 17-item Hamilton Depression Rating Scale or the 16-item Quick Inventory of Depressive Symptomatology rating scale. A *P* value of < 0.05 was considered nominally statistically significant [9, 34, 35]. See the Supplementary Text for details.

## Results

### ERICH3 gene structure and transcript variants

**Fig. 1:**
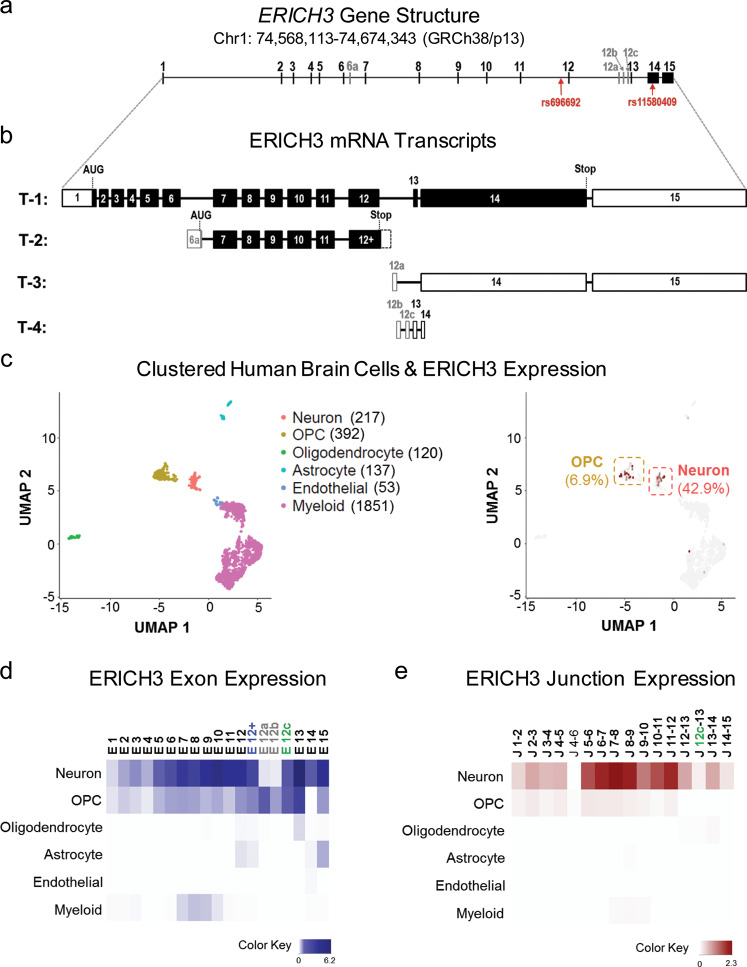
*ERICH3* gene structure and mRNA transcripts in human brain. **a** Depiction of ERICH3 gene structure based on its physical location on chromosome 1 (GRCh38/p13). ERICH3 exons were numbered from 1 to 15 based on its reference mRNA transcript (Transcript ID: ENST00000326665). Alternative exons that map to intron 6 and intron 12 of the reference mRNA transcript were named with the intron number followed by a letter of the alphabet. The positions of the rs696692 and rs11580409 SNPs are also labeled. **b** ERICH3 transcript variants (T-1 to T-4) annotated by *Ensembl*. Exons depicted as solid boxes represent open-reading frames. Untranslated regions have been depicted as empty (white) boxes. Translation start codons and stop sites have been labeled as “AUG” and “Stop,” respectively. **c** Clusters of human brain cells were assigned cell types based on the expression of cell-type-specific marker genes quantified by single-cell RNA-seq. Each dot represents a single cell. Cell types have been color-coded and the number of sequenced cells of each cell type is listed in parentheses (left). ERICH3-positive cells were color-coded based on ERICH3 expression levels, and the ERICH3-negative cells are shown as gray dots. Neurons and oligodendrocyte progenitor cells (OPC) were two cell types that included the most ERICH3 positive cells. Percentages of ERICH3-positive cells in these two cell types are presented in parentheses (right). **d** Heat map of ERICH3 exon (“E”) expression in each CNS cell type. Darker color represents higher mRNA level. Exon 6a was not detected in any of the CNS cells. **e** Heat map of ERICH3 exon junction (“J”) expression. Darker color represents higher expression. In addition to the “canonical” exon junctions, a junction of alternative exon 12c to exon 13 (“J 12c-13”) was detected at low abundance in neurons.

**Fig. 2:**
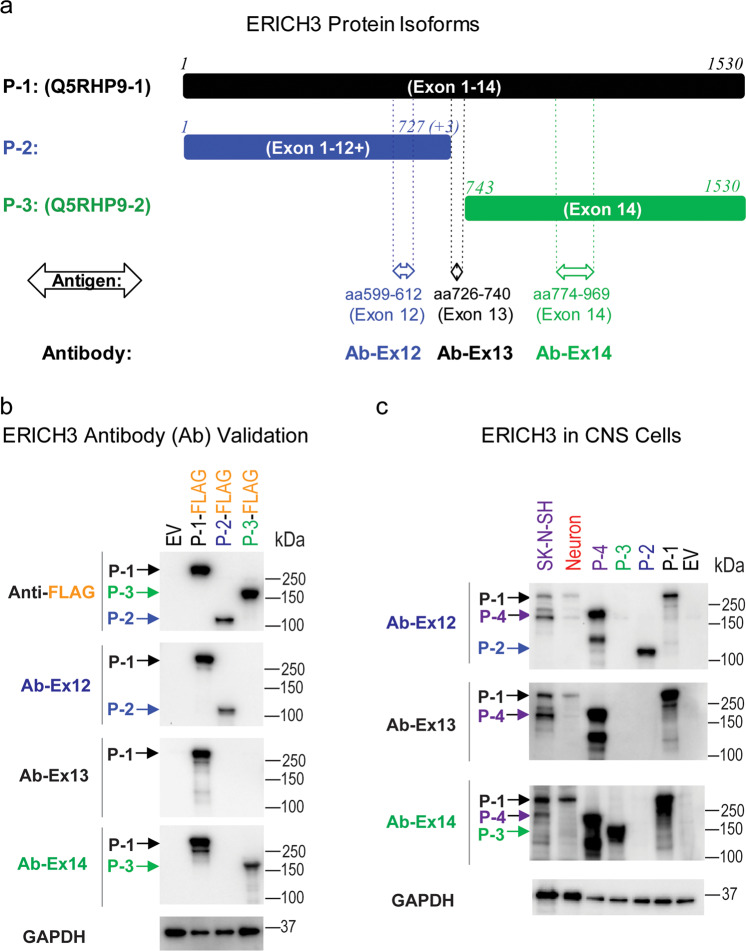
ERICH3 protein characterization. **a** Schematic depiction of ERICH3 protein isoforms. The “canonical” protein isoform encoded by the reference mRNA transcript is designated “P-1.” The UniProtKB protein ID for this isoform is “Q5RHP9-1.” The protein coding exons corresponding to each protein isoforms are shown in parentheses. Numbers of the first and last amino acids for each isoform, matching to the number of amino acids in the “canonical” protein sequence are listed above each schematic representation. “P-2” represents a putative protein isoform corresponding to the mRNA transcript variant that ends at exon 12+ in neurons. “P-3” is encoded by exon 14 which is designated “Q5RHP9-2” in UniProtKB. Antigen sequences and corresponding antibodies that target different regions of the ERICH3 protein are shown below each depiction. **b** Western blots which validate ERICH3 antibodies. ERICH3 protein isoforms fused with FLAG-tags were overexpressed in HEK293T cells and those protein lysates were subjected to Western blots using different ERICH3 antibodies. Anti-FLAG antibody was used to confirm that the FLAG-tagged ERICH3 isoforms had been successfully overexpressed. ERICH3 antibodies that target different regions of the amino acid sequences performed as expected in Western blot. All three antibodies detected P-1 as well as their specifically targeted isoforms. GAPDH were “blotted” as internal control. **c** Western blots performed using validated ERICH3 antibodies to study protein lysates from human primary neurons and SK-N-SH neuroblastoma cells, with nontagged, cDNA-overexpressed ERICH3 isoforms used as a control. “P-4” is encoded by exons 12 to 14 which corresponds to a transcript variant identified in neuroblastoma cells. Figure shown represents result from triplicate experiments.

The *ERICH3* gene structure is depicted schematically in Fig. 1a. The Ensembl human genome assembly annotated five ERICH3 mRNA transcripts. The reference transcript (ENST00000326665) consisted of fifteen exons (Fig. 1b, T-1), with exon 14 encoding half of the open reading frame. Another Ensembl protein-coding ERICH3 transcript variant (ENST00000420661) consisted of seven exons (Fig. 1b, T-2), with the first exon (numbered as “6a” in Fig. 1b) mapping to intron 6, and with other exons having sequences identical to exons 7 to 11 of the T-1 transcript. The final exon of this transcript, numbered as “12+” in Fig. 1b, contained an additional 304 nucleotides which included a stop codon and a 3’-untranslated region. Other variant transcripts annotated by Ensembl were predicted to encode either long non-coding RNAs (lncRNA) or nonsense mediated decay transcripts. Two “lncRNA” transcripts (Fig. 1b**;** T-3 and T-4), which initiated transcription from intron 12, had initial exons numbered as 12a–12c. Although RNA-seq indicated that ERICH3 was highly expressed in a variety of brain tissues [20], exactly which ERICH3 transcript(s) was/were most highly expressed in the CNS was unclear.

To determine which human brain cell type(s) expressed ERICH3, we used information from two single-cell RNA-seq (scRNA-seq) datasets generated using surgically obtained human brain tissue [27, 28]. Those CNS cells were “clustered” and cell types were assigned on the basis of mRNA expression levels for marker genes (see Supplementary Fig. S1a) [29, 30] as depicted graphically in Fig. 1c (left). ERICH3 was expressed in neurons and in oligodendrocyte progenitor cells, but predominantly in neurons, e.g., 43% of neurons were ERICH3 positive, a much higher percentage than in any other CNS cell types (Fig. 1c, right; Supplementary Fig. S1b). Using the scRNA-seq data, we generated heat maps depicting RNA expression of ERICH3 exons (Fig. 1d) and exon junctions (Fig. 1e) in CNS cells. As shown in Fig. 1d, e, a majority of exon and junction reads in neurons aligned to reference ERICH3 transcripts (Fig. 1b, T-1). Our results were similar when we used an independent scRNA-seq study performed with human cerebral cortical samples which showed that ERICH3 was broadly expressed in inhibitory and excitatory neurons but not in non-neuronal cells [36] (Supplementary Fig. S2a). ERICH3 was also reported to be expressed in serotonergic and dopaminergic neurons as shown in a scRNA-seq study performed with human fetal midbrain samples [37] (Supplementary Fig. S2b). To further validate these observations, we next characterized ERICH3 protein isoforms that were expressed in neurons.

### ERICH3 protein isoforms

**Fig. 3:**
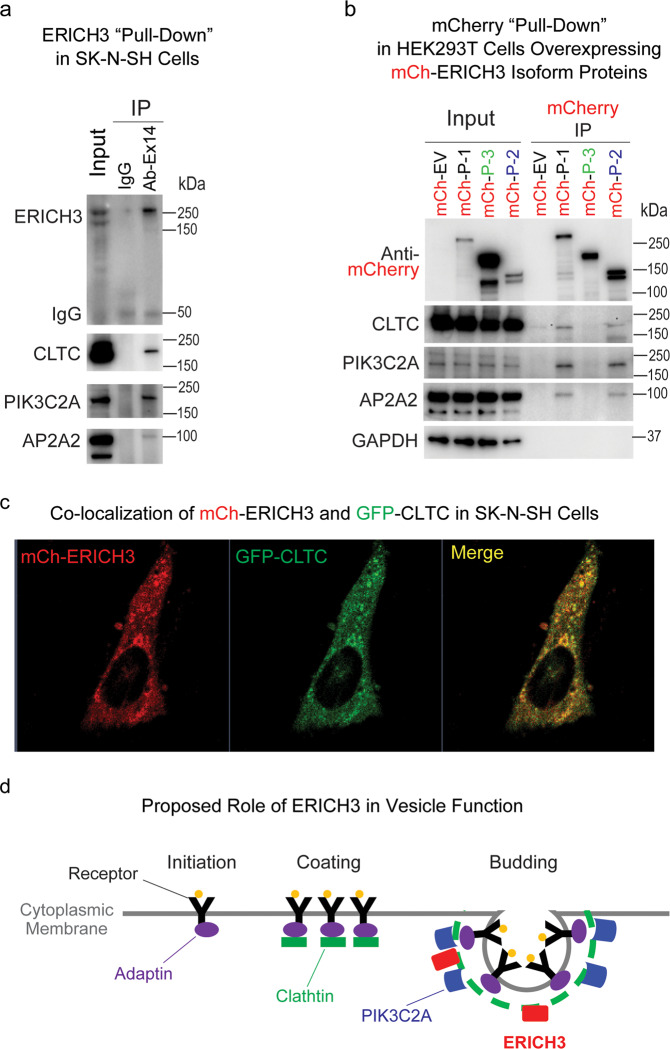
ERICH3 interacts with proteins involved in vesicular function. **a** Western blot validation of proteins that were identified by mass spectrometry in Co-IP samples precipitated by ERICH3 antibody. CLTC, AP2A2, and PIK3C2A, three proteins that play a role in vesicle biogenesis, could be “pulled-down” by ERICH3 antibody but not by control IgG in SK-N-SH neuroblastoma cells. Figure shown represents results from triplicate experiments. **b** ERICH3 protein isoforms fused with mCherry (mCh) were overexpressed in HEK293T cells and pulled-down by anti-mCherry antibody (mCherry IP). CLTC, AP2A2, and PIK3C2A were “pulled-down” by P-1 and P-2 but not P-3, indicating that the interaction between ERICH3 and those three proteins was not taking place at amino-acid sequences that mapped to P-3, a portion of the protein encoded by exon 14. Figure shown represents results from duplicate experiments. **c** Coexpression of fluorescent protein-tagged ERICH3 (mCh-ERICH3) and CLTC (GFP-CLTC) in SK-N-SH cells made it possible to visualize their cellular localization and track their movements in living cells (Supplementary Video 1). Magnification is ×100. **d** Proposed role for ERICH3 in vesicle function. The schematic is not meant to propose a specific site for ERICH3 in the structure of the vesicle.

The scRNA-seq data suggested the presence of three ERICH3 mRNA transcripts in neurons (Fig. 1d, e), transcripts that could potentially be translated into three protein isoforms (Fig. 2a, P-1 to P-3). To test that possibility, we performed Western blot analysis to detect ERICH3 protein isoforms expressed in human primary cortical neurons. To obtain control ERICH3 proteins, cDNA constructs for the three putative ERICH3 isoforms shown in Fig. 2a were cloned. We then used three ERICH3 antibodies that we created (Ab-Ex12, Ab-Ex13, and Ab-Ex14) to differentiate the ERICH3 protein isoforms (see Fig. 2a). Those same three ERICH3 isoforms, “tagged” with FLAG-epitopes, were also overexpressed in HEK293T cells, and the HEK293T lysates were used to validate our three ERICH3 antibodies which, as anticipated, differentiated ERICH3 protein isoforms based on Western blot results (Fig. 2b). When those antibodies were applied to protein lysates from human primary cortical neurons, an ERICH3 protein band pattern that matched the molecular weight (MW) of the “canonical” ERICH3 protein (P-1) was observed in human neurons (Fig. 2c). The P-1 isoform band was also detected in preparations from SK-N-SH neuroblastoma cells. In addition to P-1, a band that ran between P-1 and P-3, namely P-4, was identified in SK-N-SH cells (Fig. 2c). P-4 was assumed to be encoded by exons 12 to 14, a transcript variant identified in neuroblastoma cells [38].

The “canonical” ERICH3 protein (P-1) was predicted to have an MW of 168.5 kD, but a band at ~250 kD was observed by Western blot (Fig. 2b). Experimentally, we found that this striking difference (>80kD) between predicted and observed MWs was unlikely to result from post-translational modification such as glycosylation or phosphorylation (see Supplementary Fig. S3). Previous studies have reported that proteins with a high content of glutamate (E) and/or aspartate (D), amino acids with acidic side chains, can display higher apparent MW values during Western blot analysis than would be predicted [39], and an equation to estimating the observed MW for E/D-rich proteins has been published [39]. When that equation was used to calculate MW values for ERICH3 isoform proteins, the calculated MW values were much closer to those that we observed by Western blot analysis (Supplementary Table S2). Therefore, it is possible that the “excessive” MW values which we observed may have resulted from the high content of glutamate (E) in ERICH3. Of note, the observed MW for the “C-terminal” ERICH3 isoform P-3 which is about half the size (788 aa) of ERICH3 isoform P-1, was very close to 165.5 kD, the predicted MW of the ERICH3 P-1 isoform (Fig. 2b), thus making it difficult to distinguish these two isoforms by Western blot. However, as suggested subsequently, these two isoforms may have different functions.

### ERICH3 interaction with proteins involved in vesicular function

After characterizing the ERICH3 transcripts and proteins expressed in neurons, we next attempted to identify possible ERICH3-interacting proteins by Co-IP assays. Proteins that were “pulled-down” were separated by SDS-PAGE and visualized by silver staining (Supplementary Fig. S4). Protein bands were then cut from the gels for MS analysis. Ranked by the Sum Intensity of peptides that were quantified by MS, the “top” 50 candidate proteins identified in ERICH3 pull-down samples are listed in Supplementary Table S3. Several of those candidates proteins are known to play a role in vesicle biogenesis and/or function, including clathrin heavy chain (CLTC), adaptor related protein complex 2 subunit alpha 2 (AP2A2), AP2 associated kinase 1 (AAK1), phosphatidylinositol-4-phosphate 3-kinase catalytic subunit type 2 alpha (PIK3C2A), and myosin heavy chain 10. Those five proteins are all known to participate in vesicle formation and function [40–44]. Other proteins observed during Co-IP included TMPO, LMNA, LIMCH1, TMOD1, and POF1B, proteins that play a role in the cytoskeleton and mitosis, processes in which clathrin is also known to be involved [45, 46]. Finally, the two most abundant proteins precipitated by ERICH3 antibodies were ALK and CUX1, both of which are encoded by genes that carry genetic polymorphisms that have been associated with antidepressant treatment response [8, 47]. Both ALK and CUX1 are highly expressed in neurons and are known to play roles in neuronal differentiation, morphology, and function [48–55].

To further validate the MS results, Western blot assays were performed using antibodies that targeted MS-identified ERICH3-interacting proteins. Because the *ERICH3* SNPs were identified by a GWAS for plasma 5-HT concentrations [15] and since 5-HT, like other monoamine neurotransmitters, is stored in vesicles, we focused our subsequent studies on proteins known to be involved in vesicular trafficking and/or function (Supplementary Table S3). Three proteins, CLTC, PIK3C2A, and AP2A2, all of which could be precipitated by ERICH3 antibody from SK-N-SH cells, were validated by Western blot analysis (Fig. 3a). Immunofluorescent (IF) costaining of ERICH3 and CLTC showed colocalization of these proteins in SK-N-SH cells (Supplementary Fig. S5). Neither protein was localized in the nucleus but rather in cytoplasm, often with colocalization to vesicle-like structures (Supplementary Fig. S5). We also observed that ERICH3 was partially colocalized with vesicular monoamine transporter 2 (VMAT2) in SK-N-SH cells (Supplementary Fig. S6). To determine whether similar interactions between ERICH3 and vesicle-related proteins might also exist in other cell lines, Co-IP assays using anti-mCherry (mCh) antibody were performed with HEK293T cells after overexpression of three mCh-tagged ERICH3 isoforms. All three of the vesicle-related proteins were pulled-down by anti-mCh antibody in HEK293T cells after the overexpression of mCh-tagged ERICH3 isoforms P-1 and P-2 but not isoform P-3 (Fig. 3b). This result suggested that ERICH3 may interact with these three proteins through its N-terminal amino acid sequence which is encoded by exons 1-12. In addition, the mCh-tagged ERICH3 (mCh-ERICH3) made it possible to visualize ERICH3 cellular location by fluorescent microscopy which showed that all of three ERICH3 isoforms were present in the cytoplasm but not the nucleus (data not shown).

In an attempt to visualize the possible interaction between ERICH3 and CLTC in living cells, mCh-ERICH3 was co-overexpressed with GFP-tagged CLTC (GFP-CLTC) in SK-N-SH cells. The overexpressed mCh-ERICH3 and GFP-CLTC proteins once again showed strong colocalization (Fig. 3c). A video tracking both of the fluorescent fusion proteins showed that yellow vesicle-like structures, labeled by mCh-ERICH3 and GFP-CLTC, were moving in the cytoplasm (Supplementary Video 1), a phenotype not observed in SK-N-SH cells coexpressing mCherry itself (empty vector) with GFP-CLTC (Supplementary Video 2).

Based on the results of Western blot analysis performed after subcellular fractionation of intracellular vesicles by density gradient centrifugation, we concluded that ERICH3 is unlikely to be an intrinsic vesicle membrane protein like vesicle-associated membrane protein 2 [56] and synaptophysin [57], but rather that it appears to be a cytosolic and vesicle-associated protein like AP2A2 (Supplementary Fig. S7). Specifically, ERICH3 was primarily enriched in the same layer as was AP2A2, an “adaptor” protein that functions with clathrin during vesicle biogenesis [40] (Fig. 3d). In summary, this series of experiments suggested a possible role for ERICH3 in vesicular function (Fig. 3d), a function that can plausibly be associated with neurotransmitter concentrations, as tested directly in the next series of experiments.

### ERICH3 and cellular 5-HT

**Fig. 4:**
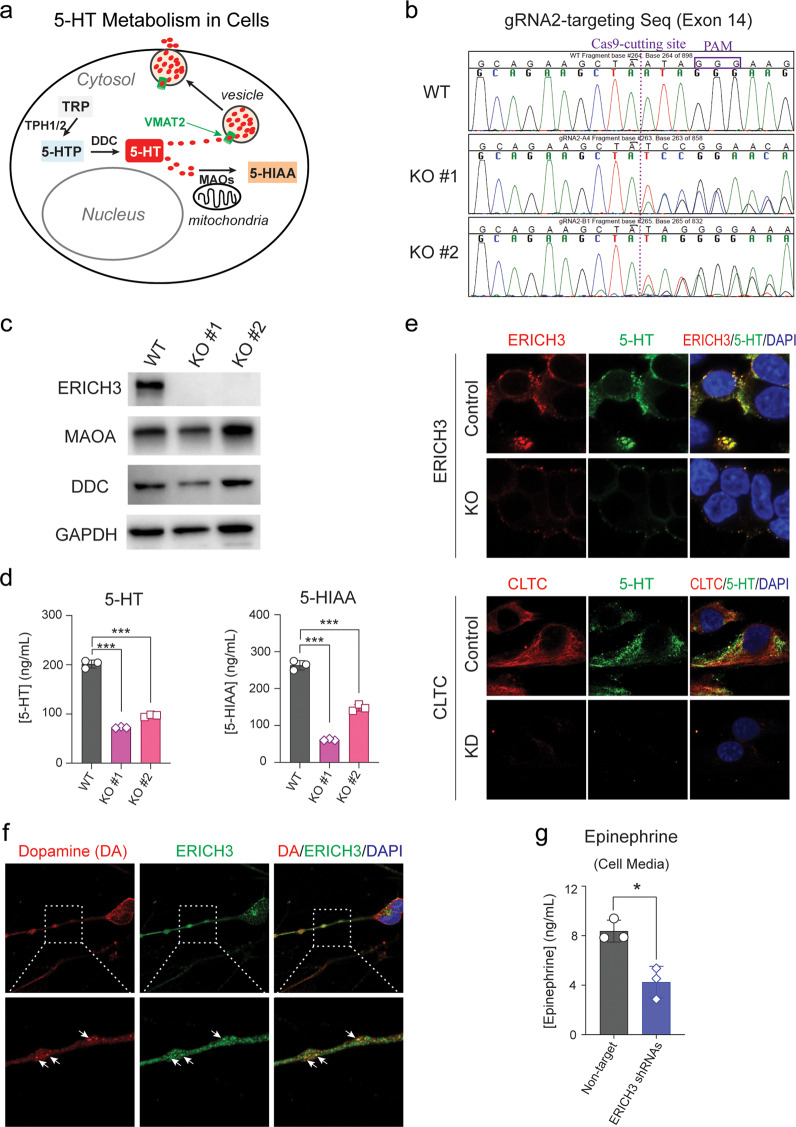
ERICH3 and monoamine neurotransmitters. **a** 5-HT is synthesized from tryptophan (TRP). Specifically, TRP is metabolized by tryptophan hydroxylases (TPH1 or TPH2) to generate 5-hydroxytryptophan (5-HTP) which can be further metabolized by aromatic-L-amino-acid decarboxylase (also known as DOPA decarboxylase, DDC) to form 5-HT. 5-HT can then be transported into vesicles by vesicular monoamine transporters (VMATs) and is released from cells by exocytosis. Vesicle-free 5-HT can be metabolized by monoamine oxidases (MAOs), which are located in the outer mitochondrial membrane to generate 5-hydroxyindoleacetic acid (5-HIAA). **b** Sanger sequencing of the *ERICH3* exon 14 region, which was targeted by ERICH3 guide RNA 2 (gRNA2) in WT SK-N-SH cells, and two single colonies edited by CRISPR/Cas9 (KO#1 and KO#2). The Cas9-cutting site is labeled by dashed line and the protospacer adjacent motif (PAM), “GGG,” is boxed. Compared to WT cells, DNA sequences on the right side of the Cas9-cutting site shifted in KO#1 and KO#2 cells, indicating that the CRISPR/Cas9 editing occurred at the “designed” site in KO#1 and KO#2 cells. **c** Western blots showing that ERICH3 protein was not detectable and that MAOA and DDC protein levels were not significantly changed in the KO#1 or KO#2 cells when compared with WT cells. **d** 5-HT (left) and 5-HIAA (right) concentrations were significantly decreased in the cell culture media of SK-N-SH KO#1 and KO#2 cells in which the *ERICH3* gene had been edited by CRISPR/Cas9. Cells were incubated with 40 µM 5-HTP for 6 h since SK-N-SH cells express neither TPH1 nor TPH2. Data are mean ± s.d. (*n* = 3), with statistical significance determined by Dunnett’s test denoted as ***P < 0.001. **e** Immunofluorescent (IF) costaining of ERICH3 and 5-HT showing the colocalization of ERICH3 with 5-HT in control SK-N-SH cells. In ERICH3 KO cells, 5-HT was hardly to detect by IF under the same conditions used to study control cells (upper panel). KD of CLTC resulted in a similar effect on 5-HT as did KO of ERICH3 (lower panel). Magnification is ×150. Figure shown represents results from duplicate experiments. **f** Costaining of ERICH3 and dopamine (DA) in human iPSC-derived dopaminergic neurons (hDN) showing the colocalization of ERICH3 with DA in neurites. Magnification is ×60 for upper panel and 300× for lower panel. Figure shown represents results from duplicate experiments. **g** Epinephrine, an end product of the dopamine metabolizing pathway was decreased in cell culture media for hDN 24 h after transfection with lentivirus packaged with ERICH3 shRNAs. Neither DA nor norepinephrine could be detected after 24 h incubation. Data are mean ± s.d. (*n* = 3), with statistical significance determined by unpaired *t*-test deno*t*ed as *P < 0.05.

To help determine how ERICH3 might influence 5-HT concentrations, both within the cell and in cell culture media, we performed a series of experiments using SK-N-SH neuroblastoma cells. 5-HT metabolism in these cells is depicted schematically in Fig. 4a. SK-N-SH cells do not express either TPH1 or TPH2, but they do express DDC and MAOA (see Supplementary Fig. S8) so they can potentially synthesize 5-HT from 5-HTP and then metabolize it to form 5-HIAA. Assessment of the dynamics of 5-HTP metabolism in SK-N-SH cells by assaying 5-HT and 5-HIAA in cell culture media showed that 5-HTP was metabolized to form 5-HT and 5-HIAA as early as after 3-h of incubation (Supplementary Fig. S9a). In cells cotransfected with Cas9 plasmid and two ERICH3 guide RNAs (gRNAs), ERICH3 protein level was decreased when compared with that in nontarget control cells (Supplementary Fig. S9b), indicating that *ERICH3* KO occurred in a portion of those transfected cells. After 6 h of incubation for the cells and 12 h for the culture medium, significant decreases in 5-HT concentrations were observed for ERICH3 gRNA-transfected cells (Supplementary Fig. S9c). 5-HIAA concentrations were also decreased in the cell culture media, but they increased in cell lysates from ERICH3 gRNA-transfected cells (Supplementary Fig. S9d). The significant decreases in 5-HT concentrations in culture media for ERICH3 gRNA-transfected cells were probably not caused by 5-HT metabolism since the expression of DDC and MAOA was unchanged (Supplementary Fig. S9b). To further confirm that the observed phenotypic changes resulted from *ERICH3* KO, two single colonies in which *ERICH3* was edited by Cas9 with gRNA2 were selected. CRISPR/Cas9 editing of *ERICH3* and the protein KO in those two single colonies (KO#1 and KO#2) were confirmed by Sanger sequencing (Fig. 4b) and Western blot assay (Fig. 4c), respectively. Even more dramatic decreases in 5-HT and 5-HIAA concentrations in culture media for those two *ERICH3* KO single colonies were observed (Fig. 4d) than had been observed for the experiment shown in Supplementary Figure S9 in which a mixture of KO and non-KO cells were present. In both cases, there were not significant changes in DDC and MAOA protein levels (Fig. 4c).

Since we had observed that ERICH3 interacts with proteins such as CLTC that are involved in vesicular function (Fig. 3), we next determined whether the observed decreases in 5-HT concentrations might be relevant to the packaging or transport of 5-HT by vesicles. Cellular 5-HT was detected by immunofluorescent staining of SK-N-SH cells incubated with 5-HTP for 6 h. We observed strong colocalization of ERICH3 and 5-HT in control cells, but both were barely detectable after ERICH3 KO (Fig. 4e, upper panels). KD of CLTC had an effect on cellular 5-HT which was similar to that observed after ERICH3 KO (Fig. 4e, lower panels).

### ERICH3 in iPSC-derived dopaminergic neurons

Our results suggested that ERICH3 might regulate 5-HT concentrations, at least in part, through a role in vesicular function, a mechanism that could potentially apply to other neurotransmitters since scRNA-seq data raised the possibility of ERICH3 expression in a variety of neurons. To test the hypothesis that ERICH3 might play a similar role for other monoamine neurotransmitters, human iPSC-derived dopaminergic neurons (hDNs) were cultured for use in ERICH3 and dopamine (DA) costaining. Those cells were first characterized by immunostaining tyrosine hydroxylase (TH), the enzyme that catalyzes the rate-limiting step in catecholamine biosynthesis, and tubulin beta-3 chain (TUJ-1), a neuron-specific microtubular protein (Supplementary Fig. S10). ERICH3 was localized to hDN cell cytosol and neurites. It also colocalized with DA in vesicle-like “dots” present in neurites (Fig. 4f), perhaps representing dopamine-containing vesicles. Cell culture media was also collected for the assay of catecholamines and their metabolites. Those assays showed that the KD of ERICH3 in hDNs decreased the concentration in culture media of epinephrine, a catecholamine neurotransmitter that requires DA as a precursor (Fig. 4g). Other catecholamines, ie dopamine and norepinephrine, could not be detected in the culture media, which might be due to the presence of several types of catecholamine synthesizing cells in the culture—including cells that synthesize epinephrine.

### ERICH3 rs11580409 SNP association with antidepressant response

**Fig. 5:**
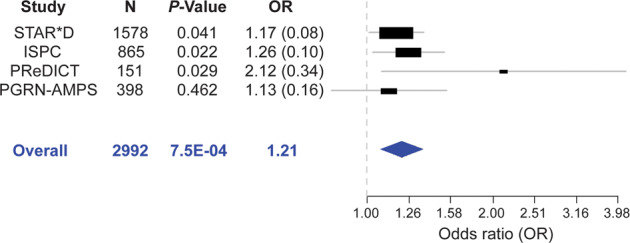
Forest plot for meta-analysis of the association of the *ERICH3* rs11580409 SNP with antidepressant treatment response. Data were obtained from four antidepressant treatment response clinical studies: Sequenced Treatment Alternatives to Relieve Depression (STAR*D), International SSRI Pharmacogenomics Consortium (ISPC), Predictors of Remission in Depression to Individual and Combined Treatments (PReDICT), and Pharmacogenomics Research Network Antidepressant Medication Pharmacogenomic Study (PGRN-AMPS). MDD patients in the STAR*D, ISPC and PGRN-AMPS studies were treated with an SSRI, either citalopram or escitalopram. PReDICT patients were treated with escitalopram or duloxetine, an SNRI. Response was defined as ⩾50% reduction in depression score as measured by QIDS-C16 or HAM-D, dependent on the study. For each of the studies, response at the last-available time point after antidepressant treatment was included in the analysis (STAR*D at 6 weeks, ISPC at 4 weeks, PReDICT at 12 weeks and PGRN-AMPS at 8 weeks). *P* values are for the association of the ERICH3 rs11580409 SNP variant allele (C) with antidepressant response; N = number of MDD patients; OR = odds ratio with the stand errors in parentheses.

When we tested the possible association of genotype for rs11580409 with drug response in independent SSRI clinical studies, the rs11580409 variant allele (C) was significantly associated with better response in two other SSRI response GWAS, with an odds ratio (OR) of 1.17 in the STAR*D study (*N* = 1578, *P* = 0.041) and 1.26 in the ISPC study (*N* = 865, *P* = 0.022) [15] (Fig. 5). In the present study, the association of this *ERICH3* nsSNP with antidepressant response was also replicated in MDD patients enrolled in the Predictors of Remission in Depression to Individual and Combined Treatments (PReDICT) study [31, 32], in which patients were treated with either an SSRI or a serotonin–norepinephrine reuptake inhibitor (SNRI). Once again, the rs11580409 variant allele (C) was significantly associated with better response (*N* = 151; *P* = 0.029; OR = 2.12) (Fig. 5). Meta-analysis that combined samples from these four independent antidepressant studies, STAR*D, ISPC, PReDICT and PGRN-AMPS, showed an even more significant association of the rs11580409 C allele with better response with an OR = 1.21 and *a P* value of 7.5E–04 (Fig. 5).

## Discussion

When we initially identified *ERICH3* as a “top hit” in our original GWAS, it was an uncharacterized gene that was most highly expressed in human brain [20–22]. That GWAS identified an SNP signal in *ERICH3* that was associated with plasma serotonin concentrations which were themselves associated with SSRI response in MDD patients enrolled in the Mayo PGRN-AMPS SSRI trial [15]. The same *ERICH3* SNP was associated with SSRI response in MDD patients in two independent large-cohort GWA studies, STAR*D [4] and ISPC [9]. Those observations motivated us to characterize the function of ERICH3 in order to help us understand its role in antidepressant treatment response. In the present study, by analyzing published scRNA-seq data for human brain samples [27, 28] we found that ERICH3 is predominantly expressed in neurons rather than other CNS cell types (Fig. 1). The major ERICH3 protein isoform in neurons, an isoform encoded by one of several mRNA splice variants, was also identified (Fig. 2). ERICH3-interacting proteins were then identified by performing Co-IP studies. Those proteins included CLTC as well as several other proteins involved in vesicular function (Fig. 3). IF staining for ERICH3 showed a cytoplasmic localization as well as localization to vesicle-like structures, supporting a possible role for ERICH3 in vesicle function. We then demonstrated that KO of ERICH3 decreased 5-HT concentrations both in cell culture media and in cell lysates (Fig. 4, Supplementary Fig. S9). Finally, ERICH3 was also found to colocalize with dopamine in human iPSC-derived neurons (Fig. 4), and the association between *ERICH3* SNPs and antidepressant response was replicated in the PReDICT study [31, 32], in which patients were treated with either an SSRI or SNRI (Fig. 5), indicating a possible role of ERICH3 in monoamine neurotransmitter function that extends beyond 5-HT. Since we also observed that ERICH3 is expressed in glutamatergic and GABAergic neurons [36] (Supplementary Fig. S2), the vesicular function of ERICH3 that we report in the present study might also apply to neurons beyond those involved in monoamine storage and function.

Monoamine neurotransmitters, including 5-HT, are stored in vesicles and released by exocytosis into the synaptic cleft when vesicles fuse with the plasma membrane [58]. After exocytosis, synaptic vesicles undergo endocytosis and recycle for a new round of exocytosis [58]. This process of vesicle trafficking may play a role in neuropsychiatric disorders. Clathrin-mediated vesicle formation is part of the machinery for vesicle recycling [59–61]. Components of the synaptic vesicle recycling machinery have been proposed previously as potential targets for the treatment of neuropsychiatric disorders [62]. In the present study, we have shown that ERICH3 is required for the maintenance of 5-HT concentrations, a process which could involve its interactions with CLTC, AP2A2, and PIK3C2A, proteins known to be involved in clathrin-mediated vesicle formation [40–42]. These observations may explain, at least in part, the association of *ERICH3* SNPs with SSRI response that we have observed in MDD patients [15]. Of potential interest is the fact that we also found that ERICH3 interacted with ALK and CUX1 (Supplementary Table S3), both of which have been reported to have genetic polymorphisms associated with antidepressant treatment response [8, 47]. However, molecular mechanism(s) that might explain those associations remain unknown.

SSRIs target the reuptake mechanism for 5-HT by inhibiting the activity of the 5-HT transporter (SERT or SLC6A4), resulting in increased synaptic 5-HT concentrations [63–65]. This mechanism applies to both the CNS and peripheral tissues. For example, more than 99% of the 5-HT in blood is found in platelets [66, 67]. However, platelets do not synthesize 5-HT but rather utilize SLC6A4 in their membrane to take up 5-HT generated in the intestine by enterochromaffin cells [68]. As a result, plasma 5-HT concentrations may reflect platelet 5-HT which might, in turn, reflect SLC6A4 function in the platelet membrane—thus providing a measure of the effect of SSRIs that can be accessed in the periphery. A striking decrease in plasma 5-HT concentrations has been observed in MDD patients after SSRI treatment [15–18]. This might explain our [15] and others’ [17, 18] observations that larger decreases in plasma 5-HT concentration after SSRI treatment, which could be a reflection of greater drug sensitivity, are associated with better SSRI response in MDD patients. ERICH3 is also highly expressed in platelets [23, 24], and 5-HT in platelets is stored in dense granules, lysosome-related vesicles with biogenesis that is known to involve the clathrin-mediated endocytic pathway [69].

Characterization of ERICH3 mRNA transcripts and proteins in the human CNS—as reported in this study—represents a necessary step toward understanding the possible role of ERICH3 in MDD and the response of MDD to SSRI therapy. We should point out several limitations of our studies including the fact that the *P* value for association of *ERICH3* SNPs in our original GWAS for plasma 5-HT concentrations in MDD patients did not reach the genome-wide significant threshold. Validation of this association in larger cohorts will be required. In addition, our results with regard to the role of ERICH3 in 5-HT biology were obtained using SK-N-SH neuroblastoma cells, results that will also require validation using serotonergic neurons once the technology to culture that type of neuron becomes less challenging technically. Finally, the possible functional effects of ERICH3 beyond 5-HT, as suggested by its broad expression in different types of neurons, should be addressed in the course of future studies using a variety of neurons as should the effect of CRISPR/cas9 editing of the *ERICH3* rs10580409 SNP to clarify the mechanism of the effect of this SNP on *ERICH3* function.

In summary, the present study has characterized the molecular genomics and neuronal function of *ERICH3*, a novel gene studied here primarily because of its association with SSRI antidepressant response. The results of the present experiments represent a step toward understanding the functional genomics of *ERICH3*. The results also point toward future studies of the functional basis for its role in SSRI response—a role that may involve neurotransmitter vesicular function, future studies of which might contribute to our understanding of individual variation in both MDD disease risk and antidepressant treatment response.

## Supplementary information

Supplementary Figures

Supplementary Tables

Supplementary Text

Supplementary Video 1

Supplementary Video 2
