# Supplementary material for: ERICH3: vesicular association and antidepressant treatment response
Source: Mol Psychiatry. 2020 Nov 23;26(6):2415–28. doi: 10.1038/s41380-020-00940-y (PMC8141066; doi:10.1038/s41380-020-00940-y)

**Supplementary Figures (Figures S1 – S10)**

**Figure S1.** (**a**) Sub-classification of human brain cells based on the single-cell RNA-seq data. Each dot depicts a single cell. Expression levels of representative maker genes that were used for sub-classification of cells are color-coded, with a darker color representing a higher mRNA level. The expression of *STMN2* gene was used to identify neurons, *PTPRC* to identify myeloid cells, *ETNNPL* to identify astrocytes, *DCN* to identify endothelial cells, *MOG* to identify oligodendrocytes and *GPR17* to identify oligodendrocyte progenitor cells (OPC). (**b**) Percentages of ERICH3-expressing (positive) cells in each sub-classification of cell types are shown graphically. Numbers shown in parentheses above each bar graph are the number of ERICH3-positive cells/total number of sequenced cells.


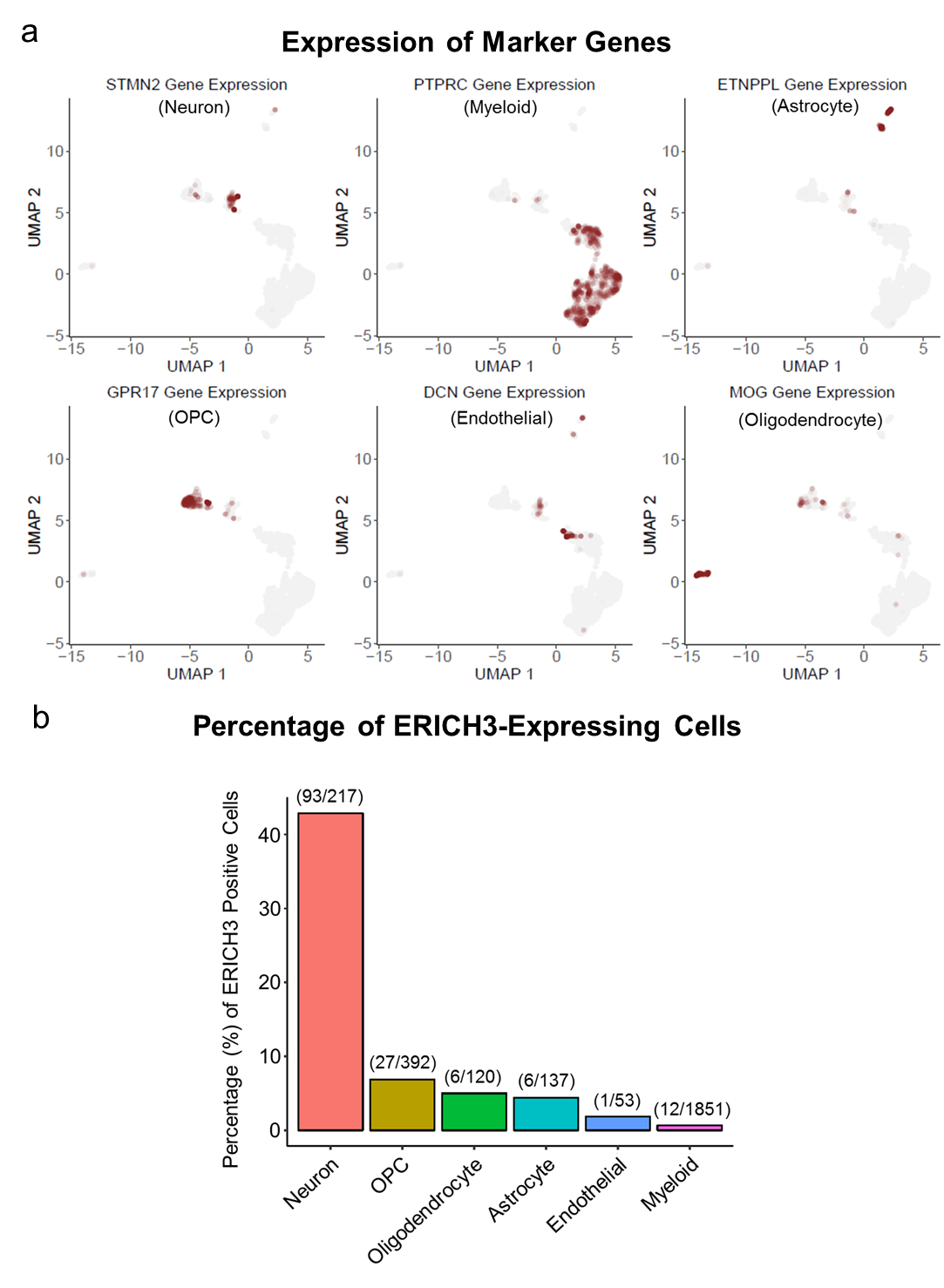


**Figure S2**. (**a**) Heat map for ERICH3 mRNA expression quantified by scRNA-seq using samples from human cerebral cortex^36^. Cells were “clustered” based on the expression of cell-type specific maker genes. Data were obtained from the Allen Human Brain Map (<http://celltypes.brain-map.org/rnaseq/human/cortex>). Specifically, scRNA-seq was performed with approximately 50,000 single cells from human cerebral cortex samples obtained from eight donors. ERICH3 was expressed predominantly in neurons, both inhibitory and excitatory neurons, rather than non-neuronal cells. (**b**) Bayesian posterior probability of expression for ERICH3 (molecules/cell). Expression levels were quantified by scRNA-seq using human fetal midbrain samples^37^. Each bar represents a sub-classified cell type. These data are publically accessible (<http://linnarssonlab.org/ventralmidbrain/>) and they indicate that ERICH3 is expressed in human serotonergic (hSert), dopaminergic (hDA1) and GABAergic (hNbGaba and hGaba) neurons. ERICH3 was also highly expressed in human radial glia-like cells (hRgl) which are neuronal progenitor cells.


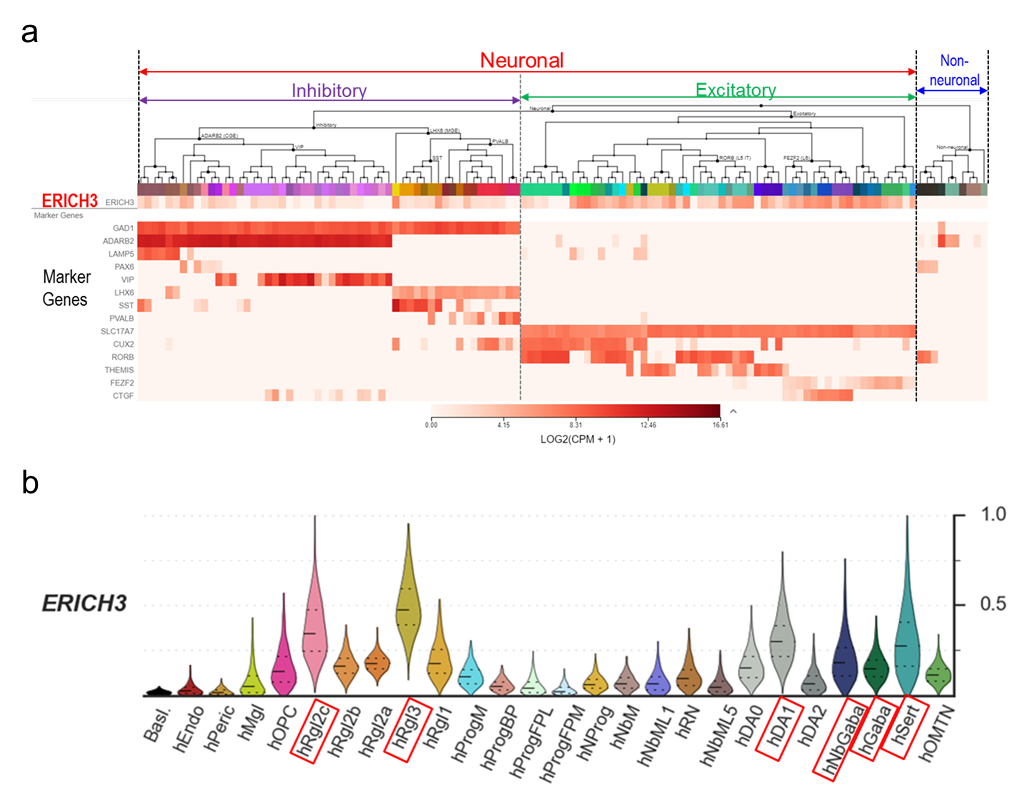


**Figure S3**. ERICH3 protein post-translational modification. “Canonical” ERICH3 protein fused with a FLAG-tag (ERICH3-FLAG) was overexpressed in HEK293T cells. Protein lysates were incubated without (-) or with (+) Protein Deglycosylation Mix II (PDglyMII), a mixture of enzymes which remove all *N*-linked and simple *O*-linked glycans as well as some complex *O*-linked glycans. The cells were also incubated without (-) or with (+) Lambda Protein Phosphatase (Lambda PP), which cleaves phosphate groups from phosphorylated serine, threonine and tyrosine residues in proteins. Western blot analysis using anti-FLAG antibody showed that neither PDglyMII nor Lambda PP incubation shifted ERICH3-FLAG bands, indicating that the higher observed than predicted MW for ERICH3 is probably not due to protein glycosylation or phosphorylation. The deglycosylation reaction was controlled by incubation of PDglyMII with a glycosylated protein, fetuin, followed by SDS-PAGE and Coomassie Blue Staining (left). Figure shown represents result from duplicate experiments.


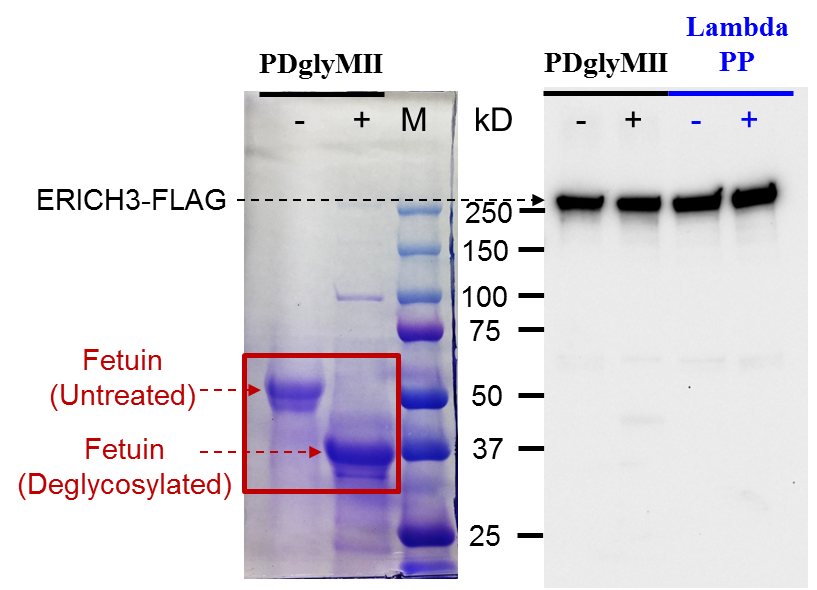


**Figure S4**. Silver staining of Co-IP protein samples “pulled-down” by ERICH3 antibody (Ab-Ex14—see **Fig. 2**). Protein samples were separated by SDS-PAGE in a 4–15% polyacrylamide gel. Each lane was cut into 3 pieces, as indicated by red dotted boxes, and was sent for mass spectrometric analysis to identify proteins. Figure shown represents result from duplicate experiments.


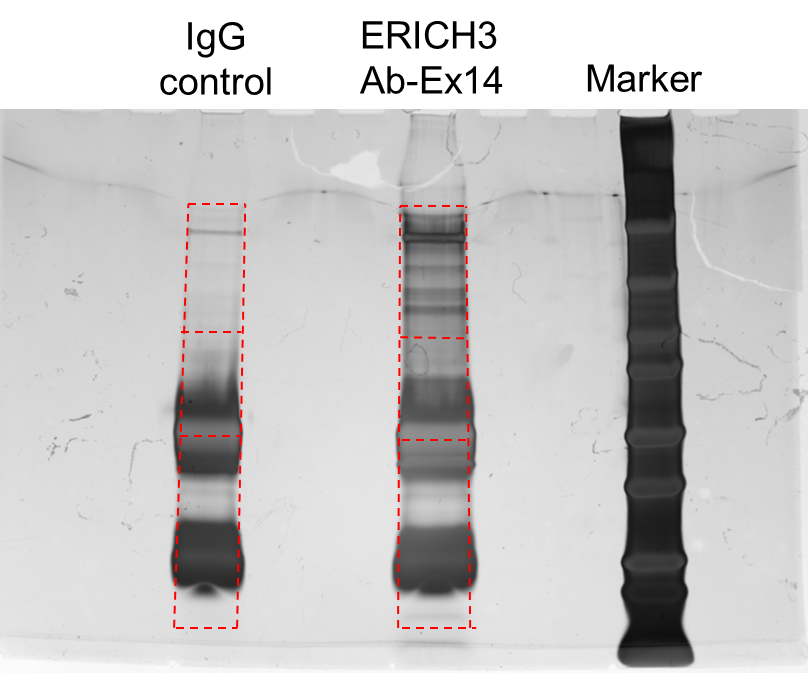


**Figure S5**. Immunofluorescent co-staining of ERICH3 and CLTC in SK-N-SH cells. Both ERICH3 (red) and CLTC (green) displayed predominantly cytoplasmic localization. Images were taken using a Zeiss LSM 780 Confocal Microscope at 100× magnification. Figure shown represents result from duplicate experiments.
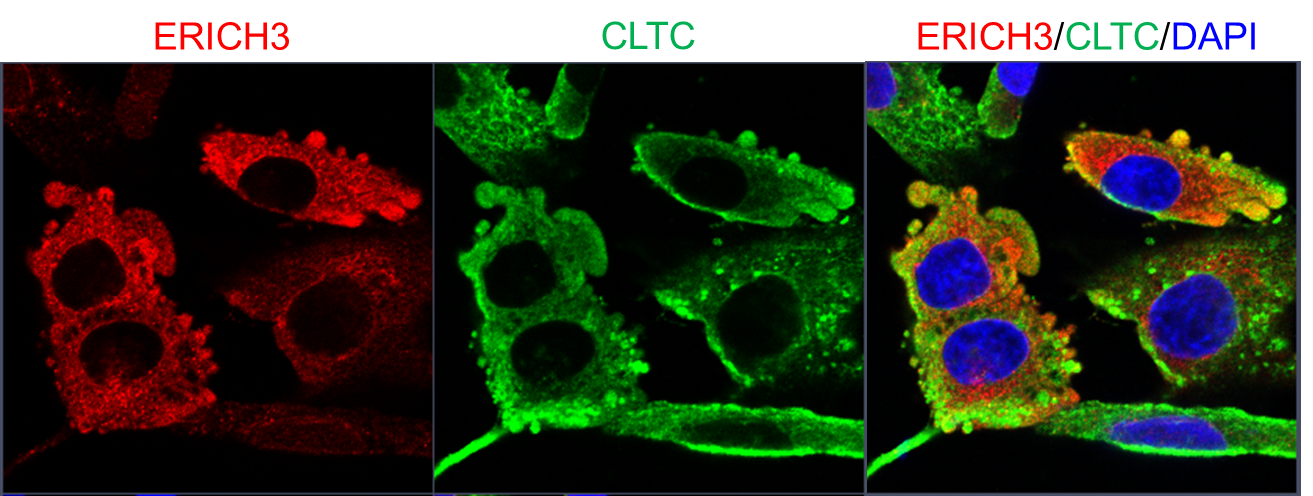


**Figure S6**. Immunofluorescent co-staining of ERICH3 and VMAT2 in SK-N-SH cells. Both ERICH3 (red) and VMAT2 (green) displayed predominantly cytoplasmic localization and co-localization was observed at plasma membranes. Images were taken using a Zeiss LSM 780 Confocal Microscope at 40× magnification. Figure shown represents result from duplicate experiments.


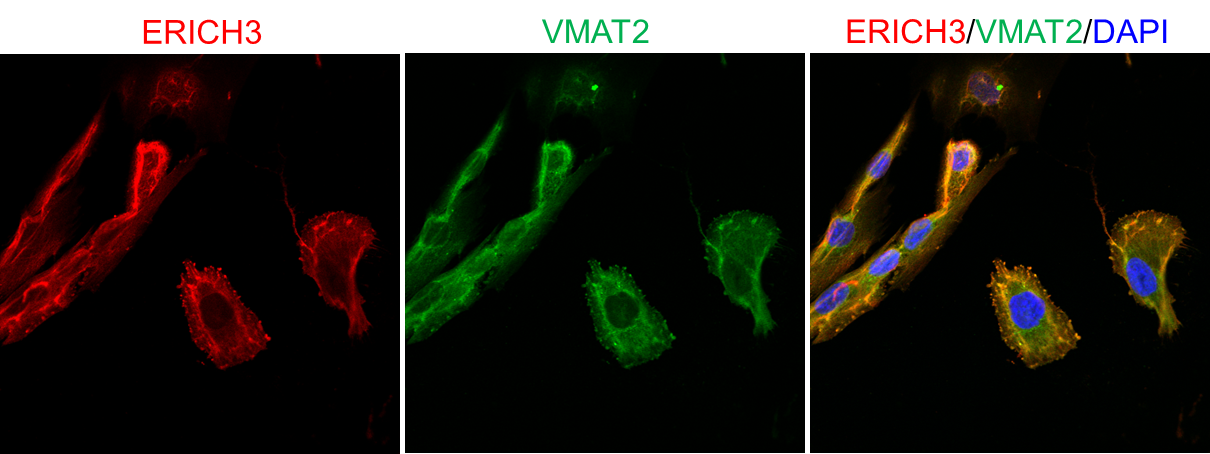


**Figure S7**. Subcellular fractionation of intracellular vesicles by differential centrifugation. (**a**) The figure represents an attempt to graphically illustrate the hypotheses that were tested: Scenario 1, ERICH3 is an intrinsic vesicular membrane protein such as vesicle-associated membrane protein 2 (VAMP2) and synaptophysin (SYP); Scenario 2, ERICH3 is not an intrinsic vesicular membrane protein but appears to interact with membrane associated proteins. SK-N-SH cells overexpressing mCh-ERICH3 were homogenized, and cell debris and nuclei were removed by low speed centrifugation. Supernatant (the post-nuclear fraction) was fractionated by density gradient centrifugation. (**b**) Depiction of the 10 layers (fractions) collected post-centrifugation for Western blot analysis. (**c**) ERICH3 was most abundant in layer #4 but the vesicle markers and intrinsic vesicle membrane proteins, VAMP2 and SYP, were most highly enriched in layer#7, suggesting that ERICH3 is not an intrinsic vesicle membrane protein. AP2A2 was most abundant in layers #3 and #4, while CLTC was most abundant in layer #8. The figure shows results obtained from duplicate experiments.


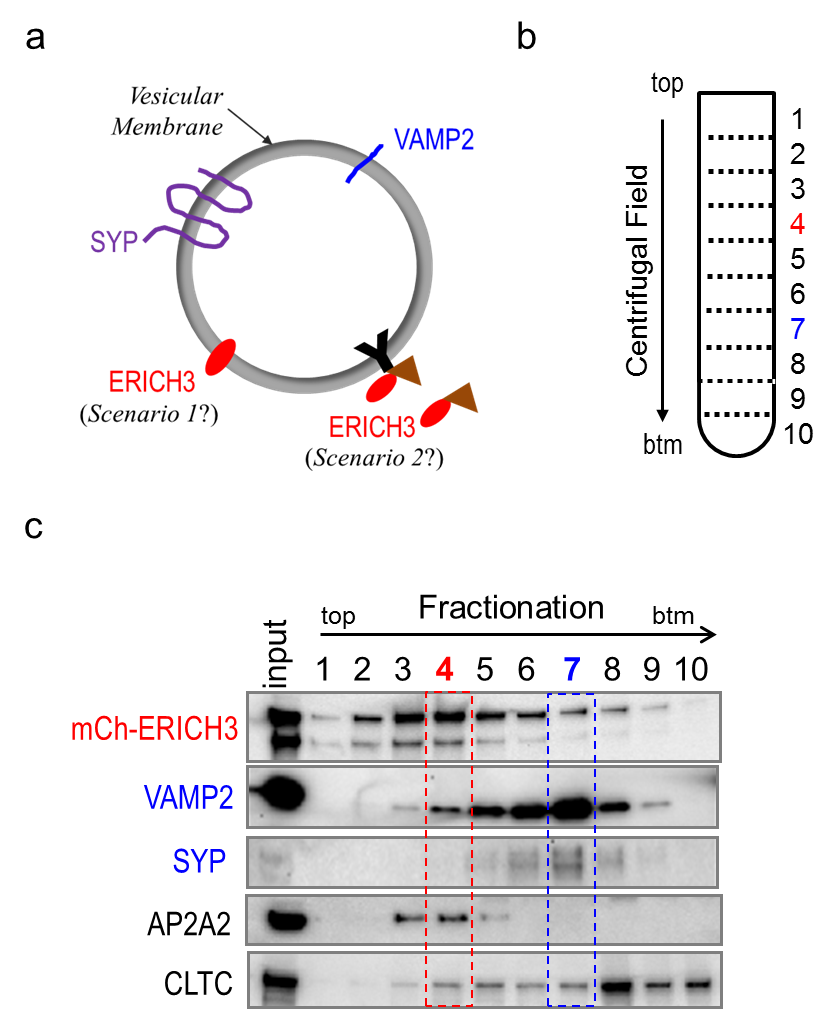


**Figure S8**. Expression of 5-HT-metabolizing pathway genes as quantified by RNA-seq in SK-N-SH cells. Data were obtained from a published RNA-seq study^38^. FPKM: Fragments Per Kilobase of transcript per Million mapped reads; TPH1 / 2: tryptophan hydroxylases 1 / 2; DDC: DOPA decarboxylase (also known as aromatic-L-amino-acid decarboxylase); MAOA/B: monoamine oxidases A/B.


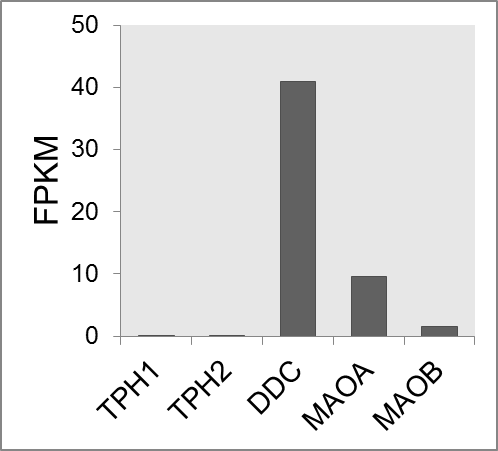


**Figure S9**. (**a**) “Dynamics” of 5-HTP metabolism in SK-N-SH cells: 5-HT and 5-HIAA were assayed in cell culture media (left) and cell lysate (right) by HPLC-ECD analysis at different time points after incubation with 40 µM of 5-HTP. Left and right y-axes are concentrations of 5-HT and 5-HIAA, respectively. No 5-HT or 5-HIAA was detected when the cells were not incubated with 5-HTP. Data is mean ± s.d. (n =4). (**b**) Western blots of protein lysates from SK-N-SH cells that were co-transfected with Cas9 cDNA plasmids and guide RNAs targeted to *ERICH3* exon7 (gRNA1) or exon 14 (gRNA2), respectively. Compared with cells transfected with non-target gRNA, ERICH3 protein levels in cells transfected with gRNA1 or gRNA2 were decreased, indicating that ERICH3 was knocked-out (KO) in a portion of the transfected cells. Protein levels of DDC and MAOA were not significantly changed in the SK-N-SH cells after ERICH3 KO. Figures shown represent results from duplicate experiments. (**c**) 5-HT concentrations were significantly decreased in both cell culture media (left) and cell lysates (right) of SK-N-SH cells in which the *ERICH3* gene had been edited by CRISPR/cas9. Cells were incubated with 40 µM 5-HTP for 6 hours since SK-N-SH cells express neither TPH1 nor TPH2. Data are mean ± s.d. (n=4), with statistical significance determined by Dunnett’s test denoted as ** *P* <0.01, ****P* <0.001, and ns = not significant. (**d**) 5-HIAA concentrations were significantly decreased in the cell culture media (left) but increased in cell lysates (right) of SK-N-SH cells in which the *ERICH3* gene had been edited by CRISPR/cas9. Cells were incubated with 40 µM 5-HTP for 6 hours. Data are mean ± s.d. (n=4), with statistical significance determined by Dunnett’s test denoted as * *P* <0.05 and ns = not significant.


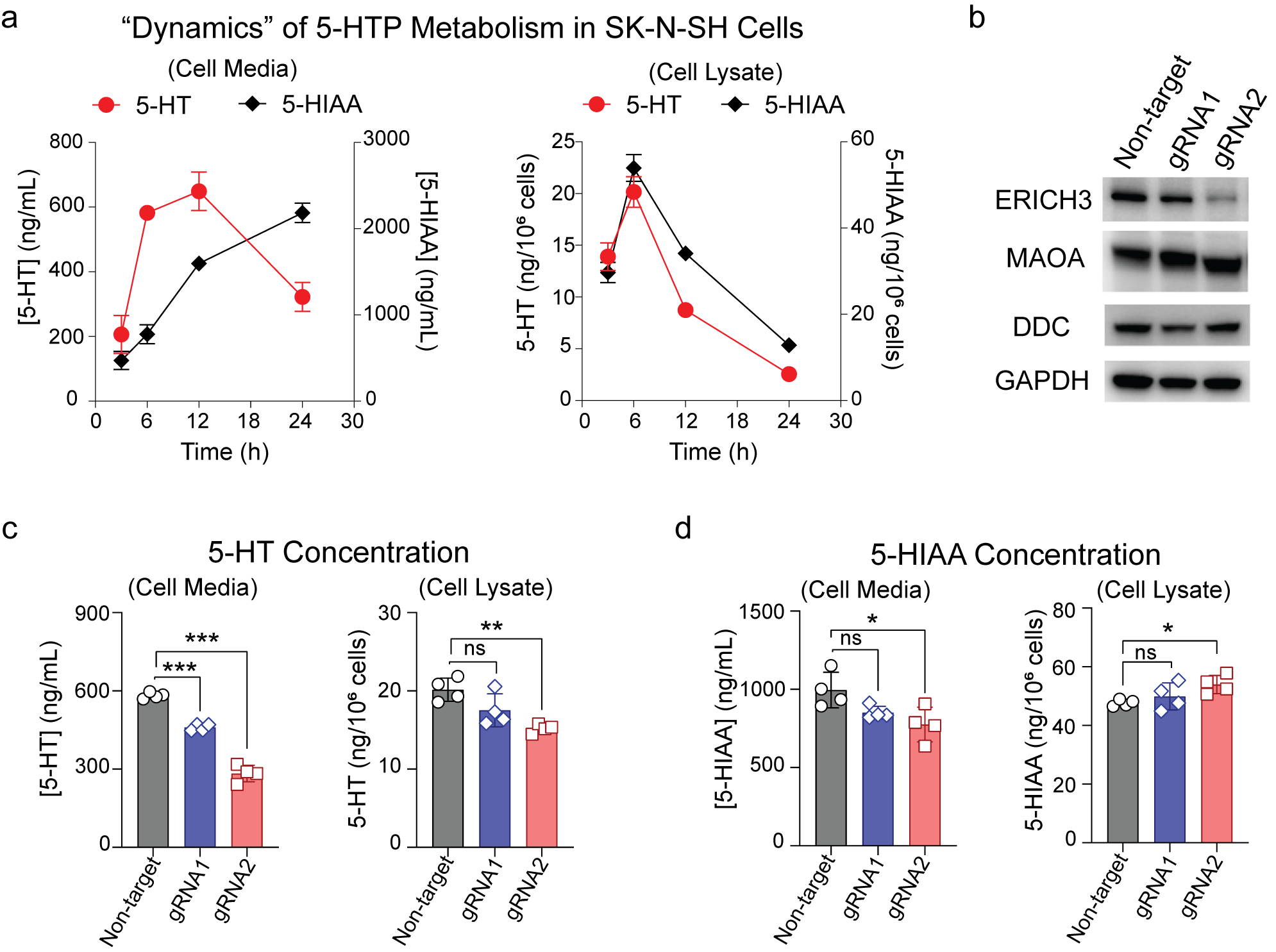


**Figure S10**. Characterization of human iPSC-derived dopaminergic neurons by immunofluorescent staining. Markers for dopaminergic neurons, tyrosine hydroxylase (TH) and dopamine (DA), were co-stained which showed that both markers were positive in certain cells (upper panels). TH was also co-stained with TJU-1, a neuron marker, which indicated that most of the cells were neurons. Images were taken using a Zeiss LSM 780 Confocal Microscope at 40× magnification. The figures shown show the results of duplicate experiments.


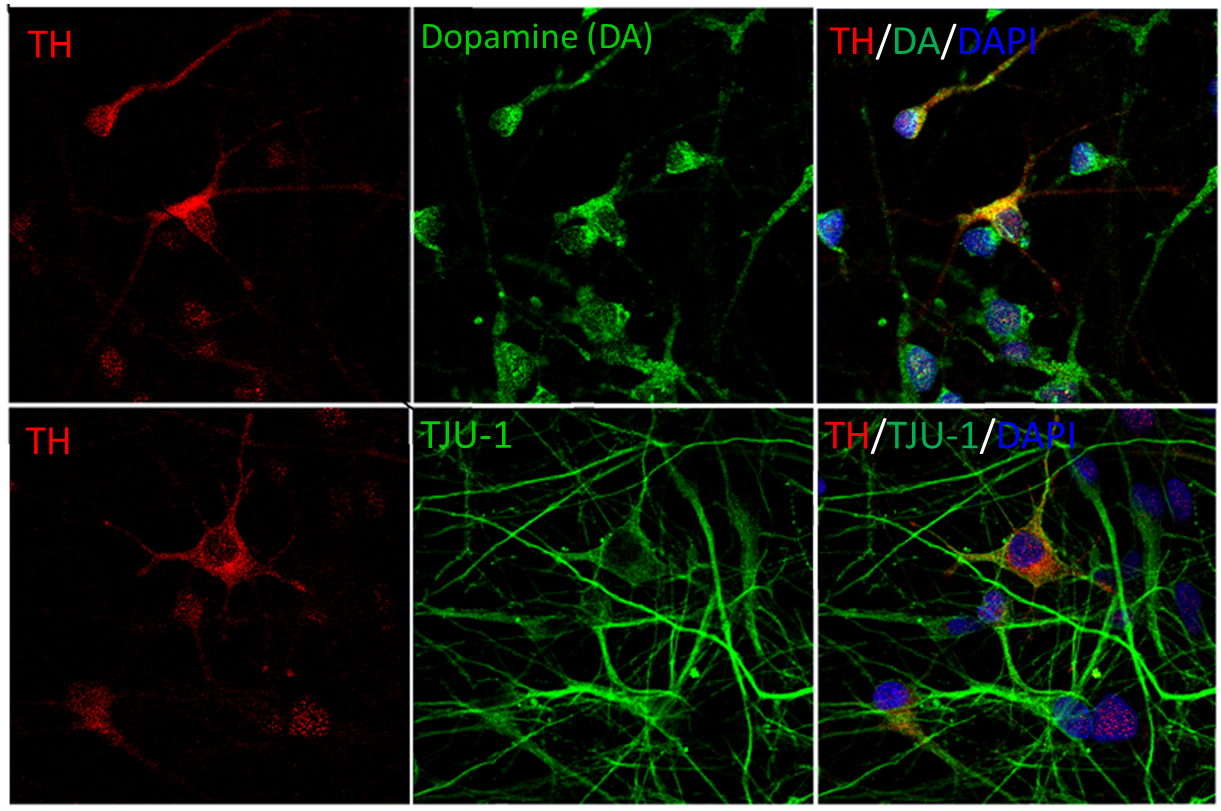

Supplement: Supplementary file 1 — Supplementary Figures [file 41380_2020_940_MOESM1_ESM.docx]
