# Supplementary material for: ERICH3: vesicular association and antidepressant treatment response
Source: Mol Psychiatry. 2020 Nov 23;26(6):2415–28. doi: 10.1038/s41380-020-00940-y (PMC8141066; doi:10.1038/s41380-020-00940-y)
Supplement: Supplementary file 2 — Supplementary Tables [file 41380_2020_940_MOESM2_ESM.docx]

| **Supplementary Table S1. Major Resources Used in this Study.** | | |  |  |  |
| --- | --- | --- | --- | --- | --- |
| **a**) **Primers for Sub-clones of ERICH3 Isoform Proteins.** A pCMV6-Entry plasmid sub-cloned with the ERICH3 P-1 protein open-reading frame (Catalog#: RC222445, OriGene Technologies, Inc. Rockville, MD) was used as template. Restriction enzymes, *AsiS*I and *Xho*I, were used to clone cDNA for the pCMV6-Entry vector, and *Sac*I and *BamH*I were used to clone the mCherry2-C1 vector. Enzyme sites are underlined, and translation start and stop codons are colored. | | | | | |
| ERICH3 Isoform Proteins | Backbone Plasmid (Vector) | Forward | | Reverse | |
| P-2 | pCMV6-Entry | GATCCGGTACCGAGGAGAT | | atgc*CTCGAG*ACGGCCATACTTTCCACCTTCCTCCAACC | |
| P-3 | pCMV6-Entry | gcac*GCGATCGC*CATGAATTTCATGGTGGATGAAAC | | atgc*CTCGAG*GACCTGCACGTTGTTGGG | |
| P-4 | pCMV6-Entry | gcac*GCGATCGC*CATGAAAACTGCTTCATCAACCTC | | atgc*CTCGAG*GACCTGCACGTTGTTGGG | |
| P-1 | mCherry2-C1 | tact*GAGCTC*TCATGAGCCATTCTCACCCC | | acat*GGATCC*TACGTGACCTGCACGTTGTTG | |
| P-2 | mCherry2-C1 | tact*GAGCTC*TCATGAGCCATTCTCACCCC | | acat*GGATCC*TAACGGCCATACTTTCCACCTTCCTCCAACC | |
| P-3 | mCherry2-C1 | tact*GAGCTC*TCATGAATTTCATGGTGGATGAAAC | | acat*GGATCC*TAGACCTGCACGTTGTTGGG | |
| P-4 | mCherry2-C1 | tact*GAGCTC*TCATGAAAACTGCTTCATCAACCTC | | acat*GGATCC*TAGACCTGCACGTTGTTGGG | |
|  |  |  |  |  |  |
| **b) Antibodies** |  |  |  |  |  |
| Primary Antibody: |  |  |  |  |  |
| Target | Vendor | Catalog Number | Host | Clonality | Usage (dilution) |
| ERICH3 (Ab-Ex12) | GenScript | Customized | Rabbit | Polyclonal | WB (1:5000) |
| ERICH3 (Ab-Ex13) | GenScript | Customized | Rabbit | Polyclonal | WB (1:5000) |
| ERICH3 (Ab-Ex14) | Cocalico | Customized | Rabbit | Polyclonal | WB (1:100000), IP (1:500) |
| C1orf173 (ERICH3) | ThermoFisher | [PA5-55933](https://www.thermofisher.com/antibody/product/ERICH3-Antibody-Polyclonal/PA5-55933) | Rabbit | Polyclonal | IF (1:100) |
| Anti-FLAG® | Sigma | [F1804](https://www.sigmaaldrich.com/catalog/product/sigma/f1804?lang=en&region=US) | Mouse | Monoclonal | WB (1:1000) |
| GAPDH | Novus | [NB300-221](https://www.novusbio.com/products/gapdh-antibody-1d4_nb300-221) | Mouse | Monoclonal | WB (1:2000) |
| CLTC | abcam | [ab172958](https://www.abcam.com/clathrin-heavy-chain-antibody-epr12235b-ab172958.html) | Rabbit | Monoclonal | WB (1:5000) |
| CLTC | Novus | [NB300-613](https://www.novusbio.com/products/clathrin-heavy-chain-1-chc17-antibody-x22_nb300-613) | Mouse | Monoclonal | IF (1:500) |
| AP2A2 | ThermoFisher | [PA5-65347](https://www.thermofisher.com/antibody/product/AP2A2-Antibody-Polyclonal/PA5-65347) | Rabbit | Polyclonal | WB (1:200) |
| PIK3C2A | Cell Signaling | [12402S](https://www.cellsignal.com/products/primary-antibodies/pi3-kinase-class-ii-a-d3q5b-rabbit-mab/12402) | Rabbit | Monoclonal | WB (1:1000) |
| RANGAP1 | Cell Signaling | [36067S](https://www.cellsignal.com/products/primary-antibodies/rangap1-d2t7t-rabbit-mab/36067?site-search-type=Products&N=4294956287&Ntt=rangap1&fromPage=plp&_requestid=347663) | Rabbit | Monoclonal | WB (1:1000) |
| CUX1 | Sigma | HPA003317 | Rabbit | Polyclonal | WB (1:250) |
| SEC16A | Novus | [NB100-1799](https://www.novusbio.com/products/sec16a-antibody_nb100-1799) | Rabbit | Polyclonal | WB (1:1000) |
| MTH10 | ThermoFisher | [19673-1-AP](https://www.thermofisher.com/antibody/product/MYH10-Antibody-Polyclonal/19673-1-AP) | Rabbit | Polyclonal | WB (1:2000) |
| mCherry | abcam | [ab213511](http://www.abcam.com/mCherry-antibody-EPR20579-ab213511.html) | Rabbit | Monoclonal | IP (1:125) |
| mCherry | Sigma | [SAB2702291](https://www.sigmaaldrich.com/catalog/product/sigma/sab2702291?lang=en&region=US&gclid=CjwKCAjw-bLVBRBMEiwAmKSB86kVSrn75hJzTFLNJJg9BaZJ7-72SQRoqiDPe5nT6-L0O_1vVLFNhhoCL3wQAvD_BwE) | Mouse | Monoclonal | WB (1:2000) |
| VMAT2 | Everest Biotech | [EB06558](https://everestbiotech.com/product/goat-anti-vmat2-slc18a2-antibody/) | Goat | Polyclonal | IF (1:250) |
| SYP | ThermoFisher | [MA5-11575](https://www.thermofisher.com/antibody/product/Synaptophysin-Antibody-clone-SYP02-Monoclonal/MA5-11575) | Mouse | Monoclonal | WB (1:1000) |
| VAMP2 | abcam | [ab181869](http://www.abcam.com/VAMP2-antibody-EPR12790-ab181869.html) | Rabbit | Monoclonal | WB (1:1000) |
| MAOA | abcam | [ab126751](https://www.abcam.com/monoamine-oxidase-a--mao-a-antibody-epr7101-ab126751.html) | Rabbit | Monoclonal | WB (1:1000) |
| DDC | Sigma | [D0180](https://www.sigmaaldrich.com/catalog/product/sigma/d0180?lang=en&region=US) | Mouse | Monoclonal | WB (1:1000) |
| Anti-Serotonin | abcam | [ab66047](http://www.abcam.com/serotonin-antibody-ab66047.html) | Goat | Polyclonal | IF (1:100) |
| Anti-Dopamine | Novus Bio | [NB110-2538](https://www.novusbio.com/products/dopamine-antibody-2b11_nb110-2538?utm_source=sciquest&utm_medium=referral&utm_campaign=product) | Mouse | Monoclonal | IF (1:100) |
| TH | abcam | [ab112](http://www.abcam.com/Tyrosine-Hydroxylase-antibody-ab112.html) | Rabbit | Polyclonal | IF (1:500) |
| TUJ-1 | abcam | [ab14545](https://www.abcam.com/beta-iii-tubulin-antibody-tuj-1-ab14545.html) | Mouse | Monoclonal | IF (1:500) |
| Secondary Antibody: |  |  |  |  |  |
| Target | Vendor | Catalog Number | Host | Conjugate | Usage (dilution) |
| Anti-Rabbit IgG (H+L) | ThermoFisher | [65-6120](https://www.thermofisher.com/antibody/product/Goat-anti-Rabbit-IgG-H-L-Secondary-Antibody-Polyclonal/65-6120) | Goat | HRP | WB (1:5000) |
| Anti-Mouse IgG (H+L) | ThermoFisher | [31430](https://www.thermofisher.com/antibody/product/Goat-anti-Mouse-IgG-H-L-Secondary-Antibody-Polyclonal/31430) | Goat | HRP | WB (1:5000) |
| Anti-Rabbit IgG (L) | abcam | [ab99697](http://www.abcam.com/Mouse-monoclonal-SB62a-Rabbit-IgG-light-chain-HRP-ab99697.html) | Mouse | HRP | WB (1:5000) |
| Anti-Mouse IgG (H+L) | Cell Signaling | [4408S](https://www.cellsignal.com/products/secondary-antibodies/anti-mouse-igg-h-l-f-ab-2-fragment-alexa-fluor-488-conjugate/4408?site-search-type=Products&N=4294956287&Ntt=4408s&fromPage=plp&_requestid=2731012) | Goat | Alexa Fluor® 488 | IF (1:1000) |
| Anti-Rabbit IgG (H+L) | Cell Signaling | [8889S](https://www.cellsignal.com/products/secondary-antibodies/anti-rabbit-igg-h-l-f-ab-2-fragment-alexa-fluor-594-conjugate/8889) | Goat | Alexa Fluor® 594 | IF (1:1000) |
| Anti-Rabbit IgG (H+L) | ThermoFisher | [R37116](https://www.thermofisher.com/antibody/product/Goat-anti-Rabbit-IgG-H-L-Cross-Adsorbed-Secondary-Antibody-Polyclonal/R37116) | Goat | Alexa Fluor® 488 | IF (1:1000) |
| Anti-Mouse IgG (H+L) | ThermoFisher | [R37121](https://www.thermofisher.com/antibody/product/Goat-anti-Rabbit-IgG-H-L-Cross-Adsorbed-Secondary-Antibody-Polyclonal/R37121) | Goat | Alexa Fluor® 594 | IF (1:1000) |
| Anti-Goat IgG (H+L) | ThermoFisher | [A-11055](https://www.thermofisher.com/antibody/product/Donkey-anti-Goat-IgG-H-L-Cross-Adsorbed-Secondary-Antibody-Polyclonal/A-11055) | Donkey | Alexa Fluor® 488 | IF (1:1000) |
| Anti-Mouse IgG (H+L) | ThermoFisher | [A-21203](https://www.thermofisher.com/antibody/product/Donkey-anti-Mouse-IgG-H-L-Highly-Cross-Adsorbed-Secondary-Antibody-Polyclonal/A-21203) | Donkey | Alexa Fluor® 594 | IF (1:1000) |
| Anti-Rabbit IgG (H+L) | ThermoFisher | [A-21207](https://www.thermofisher.com/antibody/product/Donkey-anti-Rabbit-IgG-H-L-Highly-Cross-Adsorbed-Secondary-Antibody-Polyclonal/A-21207) | Donkey | Alexa Fluor® 594 | IF (1:1000) |
|  | | |  |  |  |
| **c) Information for Scrambled shRNA Lentivirus** | | |  |  |  |
| shRNA Lentivirus | Vendor | Catalog Number | Target Sequence | | Exon |
| Non-targeting Control | Applied Biological Materials, Inc. | LVP015-G | GGGTGAACTCACGTCAGAA | | N.A. |
| C1orf173 (ERICH3) | Applied Biological Materials, Inc. | iV002266 | TCGGAGAAAGTGAAGAGGAAGCATCCATA | | 14 |
|  |  |  | AGGCAGAAGCTAATAGGGAAGATGATAGG | | 14 |
|  |  |  | ACGGAGGTGGTGAAACGGCAGAAACAGCC | | 14 |
|  |  |  | ACACTTCCAGTGAAGCCTGATTTCACTGA | | 14 |
|  | |  |  |  |  |
| **d) Information for Edit-R™ Guide RNAs** | | |  |  |  |
| crRNA | Vendor | Catalog Number | Target Sequence | | Exon |
| Non-targeting Control | Dharmacon | U-007501-01 | N.A. | | N.A. |
| ERICH3 gRNA1 | Dharmacon | CM-021809-02-0010 | ATGAAGTTCAGGAACTCCAT | | 7 |
| ERICH3 gRNA2 | Dharmacon | CM-021809-03-0010 | GACTGAGGCAGAAGCTAATA | | 14 |

| **Supplementary Table S2.** **ERICH3 Protein Isoforms and Their Molecular Weights (MW)** | | | | | | |
| --- | --- | --- | --- | --- | --- | --- |
| ERICH3 Isoform ^a^ | Number of AA | Percentage of D/E | pI ^b^ | MW (kD) | | |
|  |  |  |  | AA Sequence Predicted ^c^ | Western Blot Observed | Equation Calculated ^d^ |
| P-1 | 1530 | 20.9% | 4.84 | 168.5 | >250 | 209.0 |
| P-2 | 730 | 16.0% | 8.40 | 82.7 | ~110 | 92.1 |
| P-3 | 788 | 25.5% | 4.34 | 84.7 | ~150 | 115.6 |
| P-4 | 954 | 25.3% | 4.39 | 102.7 | ~200 | 139.5 |
| AA = amino acid; D = aspartate, E = glutamate; pI = isoelectric point. kD = kilo daltons; ^a^ERICH3 Isoform proteins (P-1 to P-3) that are depicted in Fig. 2a. P-4 maps to the last 954 amino acids of P-1 (from aa577 to aa1530), and is assumed to be encoded by exons 12 to 14. ^b,c^ Obtained by Compute pI/Mw tool (https://web.expasy.org/compute_pi/); ^d^ Calculated by an equation designed to predict SDS-PAGE-displayed MW for D/E-rich proteins^39^: y = 276.5x − 31.33 (x represents the percentage of acidic AAs, D and E, 11.4% ≤ x ≤ 51.1%; y represents the average ΔMW per AA). | | | | | | |

| **Supplementary Table S3**. **Mass Spectrometry Identified Protein Candidates That were "Pulled-Down" by ERICH3 antibody (Ab-Ex14)** | | | | | |
| --- | --- | --- | --- | --- | --- |
| Gene Symbol | Description | MW (kDa) | Sum Intensity Quantified by MS | | Western Blot Validation ^a^ |
|  |  |  | IgG Control | Ab-Ex14 |  |
| ALK | ALK tyrosine kinase receptor | 176.33 | NF | 3.80E+06 | Negative |
| **CUX1** | **Protein CASP OS=Homo sapiens GN=CUX1 PE=1 SV=2** | **77.41** | **NF** | **3.20E+06** | **Positive** |
| SSBP1 | Single-stranded DNA-binding protein, mitochondrial OS=Homo sapiens GN=SSBP1 PE=1 SV=1 | 17.25 | NF | 2.60E+06 | NA |
| INA | Alpha-internexin OS=Homo sapiens GN=INA PE=1 SV=2 | 55.36 | 1.90E+04 | 9.50E+05 | NA |
| EMD | Emerin OS=Homo sapiens GN=EMD PE=1 SV=1 | 28.98 | NF | 5.20E+05 | NA |
| XRCC1 | DNA repair protein XRCC1 | 69.43 | NF | 4.30E+05 | NA |
| VIM | Vimentin OS=Homo sapiens GN=VIM PE=1 SV=4 | 53.62 | 3.20E+04 | 4.00E+05 | NA |
| SEC16A | Protein transport protein Sec16A | 233.37 | NF | 3.70E+05 | Negative |
| TUBA1C | Tubulin alpha-1C chain OS=Homo sapiens GN=TUBA1C PE=1 SV=1 | 49.86 | 1.00E+05 | 3.20E+05 | NA |
| TMPO | Lamina-associated polypeptide 2, isoforms beta/gamma | 50.64 | NF | 2.40E+05 | NA |
| **ERICH3** | **Glutamate-rich protein 3** | **168.36** | **1.90E+04** | **1.40E+05** | **Positive** |
| **MYH10** | **Myosin-10 OS=Homo sapiens GN=MYH10 PE=1 SV=3** | **228.86** | **6.50E+04** | **1.40E+05** | **Positive** |
| TUBB4B | Tubulin beta-4B chain OS=Homo sapiens GN=TUBB4B PE=1 SV=1 | 49.8 | 4.70E+04 | 9.10E+04 | NA |
| JCHAIN | Immunoglobulin J chain OS=Homo sapiens GN=JCHAIN PE=1 SV=4 | 18.09 | NF | 8.40E+04 | NA |
| RPA1 | Replication protein A 70 kDa DNA-binding subunit | 68.1 | NF | 8.20E+04 | NA |
| RPL4 | 60S ribosomal protein L4 | 47.67 | NF | 2.40E+04 | NA |
| XRCC5 | X-ray repair cross-complementing protein 5 | 82.65 | NF | 7.70E+04 | NA |
| UBA52 | Ubiquitin-60S ribosomal protein L40 OS=Homo sapiens GN=UBA52 PE=1 SV=2 | 14.72 | 1.00E+04 | 7.20E+04 | NA |
| **PIK3C2A** | **Phosphatidylinositol 4-phosphate 3-kinase C2 domain-containing subunit alpha** | **190.56** | **NF** | **6.20E+04** | **Positive** |
| TUBB2B | Tubulin beta-2B chain OS=Homo sapiens GN=TUBB2B PE=1 SV=1 | 49.92 | 3.70E+04 | 5.90E+04 | NA |
| LMNA | Lamin-A/C | 74.09 | NF | 5.20E+04 | Negative |
| RPS18 | 40S ribosomal protein S18 OS=Homo sapiens GN=RPS18 PE=1 SV=3 | 17.71 | NF | 5.00E+04 | NA |
| **AP2A2** | **AP-2 complex subunit alpha-2** | **103.89** | **NF** | **4.50E+04** | **Positive** |
| HIST1H2AH | Histone H2A type 1-H OS=Homo sapiens GN=HIST1H2AH PE=1 SV=3 | 13.9 | 8.50E+03 | 4.50E+04 | NA |
| TUBB | Tubulin beta chain OS=Homo sapiens GN=TUBB PE=1 SV=2 | 49.64 | 2.80E+04 | 4.30E+04 | NA |
| RPL29 | 60S ribosomal protein L29 OS=Homo sapiens GN=RPL29 PE=1 SV=2 | 17.74 | 0.00E+00 | 4.10E+04 | NA |
| PARP1 | Poly [ADP-ribose] polymerase 1 | 113.01 | NF | 3.40E+04 | NA |
| AAK1 | AP2-associated protein kinase 1 | 103.82 | NF | 3.30E+04 | Negative |
| EEF1A1 | Elongation factor 1-alpha 1 OS=Homo sapiens GN=EEF1A1 PE=1 SV=1 | 50.11 | 1.30E+04 | 3.10E+04 | NA |
| KPNB1 | Importin subunit beta-1 | 97.11 | NF | 3.00E+04 | NA |
| **CLTC** | **Clathrin heavy chain 1** | **191.49** | **NF** | **2.60E+04** | **Positive** |
| LIMCH1 | LIM and calponin homology domains-containing protein 1 | 121.79 | NF | 2.50E+04 | NA |
| **RANGAP1** | **Ran GTPase-activating protein 1** | **63.5** | **NF** | **2.50E+04** | **Positive** |
| LTF | Lactotransferrin | 78.13 | NF | 2.30E+04 | NA |
| SREBF2 | Sterol regulatory element-binding protein 2 | 123.61 | NF | 2.30E+04 | NA |
| MYL6 | Myosin light polypeptide 6 OS=Homo sapiens GN=MYL6 PE=1 SV=2 | 16.92 | NF | 2.20E+04 | NA |
| TCF4 | Transcription factor 4 | 71.26 | NF | 2.20E+04 | NA |
| TMOD1 | Tropomodulin-1 | 40.54 | NF | 2.10E+04 | NA |
| CHD4 | Chromodomain-helicase-DNA-binding protein 4 | 217.87 | NF | 1.90E+04 | NA |
| SEC16A | Protein transport protein Sec16A OS=Homo sapiens GN=SEC16A PE=1 SV=1 | 249.33 | NF | 1.80E+04 | Negative |
| BPIFB1 | BPI fold-containing family B member 1 | 52.41 | NF | 1.70E+04 | NA |
| MCC | Colorectal mutant cancer protein | 92.97 | NF | 1.60E+04 | NA |
| DDX17 | Probable ATP-dependent RNA helicase DDX17 | 80.22 | NF | 1.50E+04 | NA |
| ECM1 | Extracellular matrix protein 1 | 60.64 | NF | 1.50E+04 | NA |
| JAKMIP2 | Janus kinase and microtubule-interacting protein 2 | 94.88 | NF | 1.50E+04 | NA |
| POF1B | Protein POF1B, actin binding protein | 68.02 | NF | 1.40E+04 | NA |
| LMO7 | LIM domain only protein 7 | 192.58 | NF | 1.00E+04 | NA |
| HNRNPM | Heterogeneous nuclear ribonucleoprotein M | 77.46 | NF | 9.00E+03 | NA |
| **CUX1** | **Homeobox protein cut-like 1** | **164.09** | **NF** | **7.60E+03** | **Positive** |
| IMMT | MICOS complex subunit MIC60 | 83.63 | NF | 6.50E+03 | NA |
| MW = molecular weight; MS = mass spectrometry; Ab-Ex14: Anti-ERICH3 antibody which has been referred in Fig. 2 and Supplementary Table S1; NF = not found, indicating that the Sum Intensity Quantified by MS is zero; ^a^ Western blot assays were performed to validate some of those candidate proteins. Protein candidates, which were detected in Ab-Ex14 “pull-down” sample but not in IgG Control in Western blot assays (as shown in Fig. 3), are labelled as “Positive” and are **bold**. “Negative” indicates that the candidate protein was not detected in Ab-Ex14 “pull-down” sample in Western blot assays. Antibodies which were used to detect candidate proteins are listed in Supplementary Table S1. NA = not applicable, which means the Western blot validation for that protein was not performed. | | | | | |
