# Supplementary material for: ERICH3: vesicular association and antidepressant treatment response
Source: Mol Psychiatry. 2020 Nov 23;26(6):2415–28. doi: 10.1038/s41380-020-00940-y (PMC8141066; doi:10.1038/s41380-020-00940-y)
Supplement: Supplementary file 3 — Supplementary Text [file 41380_2020_940_MOESM3_ESM.docx]

**Supplementary Text:**

**Materials and Methods**

***ERICH3 mRNA Expression in Human CNS at the Single Cell Level***

Two sets of single cell RNA-seq data for the human brain were downloaded from the NCBI SRA repository. The first dataset^1^ (GEO accession: [GSE67835](https://www.ncbi.nlm.nih.gov/geo/query/acc.cgi?acc=GSE67835)) included 466 cells from brain cortical tissue removed for medical refractory seizures from eight individuals. The second dataset^2^ (GEO accession: [GSE84465](https://www.ncbi.nlm.nih.gov/geo/query/acc.cgi?acc=GSE84465)) included 2498 glioblastoma adjacent cells identified as non-neoplastic from cortex of four patients. All of the data were downloaded in SRA format, converted to fastq format using the fastqdump command from sratoolkit, quality checked by fastqc, before being mapped to the human reference genome GRCh38 (hg38) using STAR software^3^. Raw counts at the gene and exon levels were called by HTSeq^4^ using default and -t exon -i exon_id --nonunique all options. Single cells with less than 1×10^5^ total RNA counts were excluded from further analysis. Exon junction reads were counted using the Outrigger package from the Star Junction mapping reads^5^. R software (<https://www.r-project.org/>) was used for all of the subsequent analyses. For single cell deconvolution and cell population identification, the pagoda2 package^6^ was employed, as described by Darmanis S. *et al*^2^, to identify the top 500 over-dispersed genes, and dimension reduction was processed by use of the UMAP technique ( [arXiv:1802.03426v2](https://arxiv.org/abs/1802.03426v2))^7^, made available to R via the reticulate and umapr packages. Cell types were then manually identified based on the expression of specific biomarkers: STMN2 (neuron), PTPRC (myeloid), ETNPPL (astrocyte), GPR17 (Oligodendrocyte progenitor cell, OPC), DCN (endothelial) and MOG (Oligodendrocyte).

To quantify relative exon abundance, annotated exons for the *ERICH3* gene from human genome assembly GRCh38 were examined. Exons with same start and end splice sites were merged, and exons with no counts or without both independent start and end splice sites were removed. Counts were further normalized by library size and exon length (fpkm).

***ERICH3 Overexpression Plasmids***

A human ERICH3 open-reading frame (ORF) plasmid was obtained from OriGene Technologies, Inc. (Rockville, MD). This plasmid (Catalog#: RC222445) had been constructed by sub-cloning the ERICH3 ORF into a pCMV6-Entry vector, which encodes a “canonical” ERICH3 protein (UniProtKB ID: Q5RHP9-1) fused with a DDK-tag (a FLAG-tag) at its C-terminus. All of other ERICH3 isoform overexpression constructs that were used in this study were cloned using this commercially available plasmid as a template. Plasmids to overexpress ERICH3 proteins without FLAG-tags were cloned by incorporating a stop codon after the codon encoding the final amino acid of the ERICH3 protein. To overexpress mCherry-ERICH3 fusion proteins, the ORFs for ERICH3 isoform proteins were sub-cloned into an mCherry2-C1 vector (Addgene plasmid#: 54563) which was a gift from Michael Davidson. PCR primers that were used to clone ERICH3 plasmids are listed in **Supplementary Table S1a**.

***ERICH3 Antibody Generation and Validation***

Different peptides that mapped to the human ERICH3 “canonical” protein (UniProtKB ID: Q5RHP9-1) were prepared to immunize rabbits for antibody generation. To generate antibody that targeted the ERICH3 peptides encoded by exon 12 (aa 599-612) and by exon 13 (aa 726-740), the corresponding peptides were synthesized and used to immunize New Zealand rabbits. Antibody was purified by antigen affinity using anti-sera obtained after the third immunization. The entire process, from peptide synthesis to antibody purification, was performed by GenScript (Piscataway, NJ). An additional antibody targeting the ERICH3 peptide (aa 774-969) encoded by exon14 was also generated. Specifically, DNA sequence that encoded aa774-969 was sub-cloned into a pGEX-4T-2 vector plasmid (GE Healthcare, [Chicago, IL](https://www.google.com/search?q=Chicago&stick=H4sIAAAAAAAAAONgVuLQz9U3MIxPMXnE6Mgt8PLHPWEpi0lrTl5jNOLiCs7IL3fNK8ksqRRS4WKDsqS4eKTgmjQYpLi44DyeRazszhmZyYnp-QCKUZtcWgAAAA)). Cloned plasmids were transformed into *E. coli* BL21(DE3) strain (Millipore Sigma, St. Louis, MO) for overexpression of GST-tagged ERICH3 antigen peptides which were then purified by using Glutathione Sepharose^®^ 4B GST-tagged protein purification resin (GE Healthcare)^8^. More than 2 mg of purified ERICH3 antigen peptides were sent to the Cocalico Biologicals, Inc (Stevens, PA) for antibody production. Antigen peptides were injected into rabbits and the anti-sera were collected for purification of ERICH3 antibodies using a Protein A column. An ERICH3 antibody which targets the peptide (aa 955-1053) encoded by exon 14 and has been used in immunohistochemistry, was commercially available from a variety of different vendors.

These ERICH3 antibodies were validated by the use of Western blot assays using protein lysates from HEK293T cells that overexpressed ERICH3-FLAG fusion proteins. Protein lysate from cells that were transfected with pCMV6-Entry empty vector (EV) was used as negative control. Validated anti-FLAG antibody (Sigma-Aldrich, St. Louis, MO) was used to confirm that the ERICH3-FLAG fusion proteins were successfully overexpressed, which served as a positive control for Western blot assays. ERICH3 antibody which detected ERICH3 protein in overexpression samples but not in EV control samples was considered functional. Antibody information, including vendor’s catalog numbers and dilutions are listed in **Supplementary Table S1b**.

***Cell Culture***

HEK293T and SK-N-SH cell lines were obtained from the American Type Culture Collection (ATCC). HEK293T cell lines were cultured in Dulbecco's Modified Eagle Medium (DMEM) with 10% FBS, and SK-N-SH cells were cultured in Eagle's Minimum Essential Medium with 10% FBS. Human iPSC-derived dopaminergic neuron progenitor cells were purchased from Axol Bioscience (Cambridge, United Kingdom; Cat#: ax0091). Those cells were differentiated from cord blood CD34+ cells of a male newborn donor. Differentiation, maintenance and maturation of those dopaminergic neuron progenitors were performed by following the manufacture’s protocol. Briefly, cells were seeded into pre-coated culture vessels at 40,000 - 60,000 cells/cm^2^, were cultured in the Differentiation Medium for 4 days with a change to fresh media every other day. On day 5 post-seeding, Differentiation Medium was replaced by Maintenance Medium and continued to maturation. Media was changed every 2 days until 35 days post seeding.

***Transient Transfection***

HEK-293T cells were transfected with cDNA constructs by using the Lipofectamine™ 3000 Transfection Reagent (ThermoFisher, Grand Island, NY). Eight hours after transfection, cell culture media was replaced with fresh media. After 24 hours of transfection, cells were harvested for extraction of total RNA and protein lysate. SK-N-SH cells were transfected with DNA plasmids or with siRNA by electroporation using the Amaxa^®^ Cell Line Nucleofector^®^ Solution V Kit (Lonza, Cologne, Germany). For each transfection, 1E-06 cells were mixed with 2µg of plasmid DNA or 100 pmol of siRNA in 100 uL of Nucleofector^®^ Solution V and were incubated in cuvettes for 5 min without air bubbles. Sample suspensions in cuvettes were then “pulsed” on a Nucleofector^®^ II Device (Lonza, Cologne, Germany) with program X-005. After addition to the cuvette of 500 µL of pre-equilibrated culture media, samples were gently transferred to culture plates and were incubated at 37 °C overnight. To knock-down ERICH3 in human iPSC-derived dopaminergic neurons, cells were infected with lentivirus packaged with scrambled ERICH3 shRNAs and with non-targeting control shRNA (Applied Biological Materials, Inc., Richmond, BC, Canada). Detailed information for these lentiviruses is listed in **Supplementary Table S1c**.

***ERICH3 Gene-editing in SK-N-SH cells***

The *ERICH3* gene in SK-N-SH cells was edited by using the Edit-R™ CRISPR-Cas9 system (Dharmacon, Lafayette, CO). This system includes three components: a Cas9 expression plasmid with puromycin-resistance (Cat#: U-005100-120), a CRISPR RNA (crRNA) or guide RNA (gRNA), and a trans-activating crRNA (tracrRNA; Cat#: U-002005) which is partially complementary to the crRNA. These three components were co-transfected into SK-N-SH cells by electroporation as described above. After co-transfection, crRNA and tracrRNA can form a crRNA/tracrRNA hybrid which acts as a guide for the Cas9 endonuclease to cut the target DNA sequence. Two ERICH3 crRNAs (see **Supplementary Table S1d**) were used to guide Cas9 to edit *ERICH3*, and a non-targeting crRNA (Cat#: U-007501-01) was used as a control. Transfected SK-N-SH cells were selected by adding 0.25 µg/mL of puromycin for 3 days. Before the selection of *ERICH3* knock-out (KO) single-colonies, gene-editing results were tested by Western blot using protein lysates from all transfected cells, a mixture of ERICH3 wildtype (WT) and KO cells. The downstream assays, 5-HT concentration and immunofluorescence (IF), would not be affected by a mixed population with a portion of ERICH3 WT cells―e.g., changes in 5-HT concentration with “mixed” cell population can be addressed when compared with control cells, and the IF assay *per se* can differentiate WT and KO cells. After confirmation of *ERICH3* gene-editing by Western blot assay, KO single-colonies were selected and their DNA editing and ERICH3 protein KO were confirmed by Sanger sequencing and Western blot assay, respectively. Concentrations of 5-HTP metabolites in media culturing *ERICH3* KO single-colonies were also measured.

***Western Blot Analysis***

Human neuron lysate (HN Lysate, Cat#:1526) was obtained from the ScienCell Research Laboratories (Carlsbad, CA). HN lysate is prepared from early passage human neurons using a modified RIPA buffer (50 mM Tris-HCl pH 7.2, 150 mM NaCl, 1% NP-40, 1mM EDTA, 1 mM EGTA, 0.4 mM PMSF, 5 μg/ml aprotinin, 5 μg/ml leupeptin, 1 μg/ml pepstatin, 1 mM Na3VO4, 5 mM NaF). Lysate concentration was determined by Beckman Coulter DU 800 spectrophotometer. Lysate was diluted and stored in Storage Buffer (Tris-HCl pH 6.8, 5% glycerol, 1% SDS, 0.002% bromphenol blue, 5% Beta-mercaptoethanol) before use. Total protein lysates from cultured cells were prepared by adding Lysis Buffer (25 mM Tris-HCl pH 7.4, 150 mM NaCl, 1 mM EDTA, 1% NP-40 and 5% glycerol), followed by centrifugation at 14,000×g for 10 mins. Protein concentrations were determined by using the Pierce BCA Protein Assay Kit (ThermoFisher; Cat#:23227).

Equal quantities of denatured protein were loaded onto 4–20% Mini-PROTEAN® TGX™ Precast Protein Gels (Bio-Rad, Hercules, CA) to separate proteins. Proteins were transferred electrophoretically from the gels to PVDF membranes (Bio-Rad, Hercules, CA) which were then blocked with 5% non-fat milk at room temperature for 1 hour. After washing with TBST, membranes were incubated with primary antibody (detailed information has been listed in **Supplementary Table S1b**), which was dissolved in 1% BSA prepared in TBST at 4°C overnight with gentle rocking. Following incubation, the membranes were washed vigorously three times in TBST buffer and were then incubated with horseradish peroxidase (HRP)-labelled secondary antibody, which was dissolved in 5% non-fat milk, at room temperature for 1 hour. The SuperSignal West Dura Extended Duration Substrate (ThermoFisher, Cat#34075), a luminol-based enhanced chemiluminescence HRP substrate, was applied to the membranes, and radiographic images were captured by use of the ChemiDoc™ Touch Image System (Bio-Rad, Hercules, CA). GAPDH protein was employed as a loading control.

***Co-Immunoprecipitation (Co-IP) and Mass Spectrometry***

Co-IP assays were performed with ERICH3 Ab-Ex14 antibody, followed by mass spectrometric (MS) analysis of the co-precipitated proteins. Because it is difficult to generate an adequate number of human neurons to obtain the quantity of protein lysate required for Co-IP studies, protein lysates from SK-N-SH neuroblastoma cells were used since SK-N-SH cells express the ERICH3 P-1 isoform, just as do human neurons (**Fig. 2c**). Co-IP was performed by using the Pierce™ Classic Magnetic IP/Co-IP Kit (ThermoFisher; Cat#:88804). Specifically, cells were lysed with ice-cold IP Lysis Buffer (pH 7.4, 0.025M Tris, 0.15M NaCl, 0.001M EDTA, 1% NP40, 5% glycerol) for 5 minutes and were then centrifuged at ~13,000 × g for 10 minutes to pellet the cell debris. Supernatant was then transferred to a new tube for protein concentration determination by using the Pierce BCA Protein Assay Kit (ThermoFisher). For each IP reaction, a total volume of 500 μL cell lysate with approximately 2000 μg of total protein was used. Cell lysates were “pre-cleaned” by incubating with 25 μL (0.25 mg) of Pierce Protein A/G Magnetic Beads at room temperature for 1 hour which removed nonspecific protein binding after discarding the beads. Pre-cleaned lysates were then incubated with specific antibody overnight at 4ºC to form the immune complex. The antigen sample/antibody mixture was then added to 25 μL of fresh Protein A/G Magnetic Beads and was incubated at room temperature for 1 hour. Beads were collected with a magnetic stand and were washed 3 times with 500 μL of fresh IP Wash Buffer. Antigen/antibody complex was then eluted by incubating with 100 μL of low-pH Elution Buffer. Eluted protein samples were denatured by adding Laemmli buffer and heating at 100°C for 5 mins. For “endogenous” ERICH3 Co-IP in SK-N-SH cells, 5 μg of ERICH3 antibody or control IgG (#2729S, Cell Signaling) were added to 2000μg/500μL of protein lysate, respectively. For Co-IP of mCherry-tagged ERICH3 isoforms in HEK293T cells, 2 μg of mCherry antibody was added to 1000μg/500μL of protein lysate. Protein lysate from HEK293T cells that had been transfected with empty vector was used as control. Western blot assays were performed to confirm IP efficiency by comparing specific antibody “pull-down” samples with IgG and input samples. Information with regard to the antibodies used in the Co-IP and Western blot analyses are listed in **Supplemental Table S1b**.

To identify ERICH3-interacting proteins in SK-N-SH cells, IP samples were loaded on 4–15% Mini-PROTEAN® TGX™ Precast Protein Gels (Bio-Rad, Hercules, CA) to separate proteins. Protein bands were visualized by silver staining and then were cut from the gels for mass spectrometric analysis. Mass spectrometry and the results analysis were done by the Taplin Biological Mass Spectrometry Facility at Harvard Medical School (Boston, MA).

***Subcellular Fractionation***

Subcellular fractionation of intracellular vesicles was performed by density gradient centrifugation, following a published protocol (<https://bio-protocol.org/e1571>). Approximately 2.0 × 10^7^ SK-N-SH cells which were stably expressing mCherry-tagged ERICH3 were collected and re-suspended in 450 µL of ice-cold homogenization solution. Cells were homogenized by 80-100 passages through a 29-gauge needle until the cells were roughly 70-80% trypan blue positive. Homogenates were centrifuged at 1,000 × g for 5 min at 4 °C to remove cellular debris. Whole supernatant was collected for density gradient centrifugation. The gradient solution was made by layering 360 µl of 30%, 23%, 17%, 11%, and 5% OptiPrep (Sigma, St. Louis, MO) sequentially in a centrifuge tube. 300 µL of the supernatant was loaded on top of the gradient solution and was centrifuged for 4 hours at 130,000 × g at 4 °C. 200 µL aliquots per fraction were harvested from top to bottom of the centrifuge tube, which made a total of 10 fractions. Each fraction was transferred to a 1.5 mL centrifuge tube and stored at -80 °C until it was used for Western blot assays.

***Quantification of 5-HT and Dopamine Metabolites***

High-performance liquid chromatography-electrochemical detection (HPLC-ECD) was utilized to assay 5-HT, dopamine and their metabolites. Cell culture media were centrifuged at 14, 000 × g for 10 mins to remove any cells or cellular debris before HPLC assay. SK-N-SH cell culture media was collected after the cells were incubated with 40 µM of 5-HTP, at a series of times from 3 ~ 24 hours. Approximately 4 × 10^6^ cells were collected for the measurement of 5-HT inside of cells. Samples were prepared from cell lysates with perchloric acid (PCA) deproteinization. Briefly, PCA at a concentration of 1 M was added to cell lysates to denature and precipitate proteins. After centrifugation (14, 000 × g, 2 mins at 4ºC), supernatant was collected and neutralized by adding ice-cold KOH (2M). Deproteinized samples were obtained by collecting supernatant after an additional centrifugation (14, 000 × g, 15 mins at 4ºC). 50 μL of sample was injected and analyzed on a Shimadzu UFLC System (Shimadzu Corporation, Kyoto, Japan), equipped with a CBM-20A controller, LC-20AD pump, coupled with an UltiMate™ 3000 ECD-3000RS Electrochemical Detector (Thermo Scientific, San Jose, CA). Chromatographic separation was achieved on a Shimadzu C18 reversed-phase column 150 × 4.6 mm, 3 μm particle size (Shimadzu Corporation, Kyoto, Japan). The mobile phase was degassed as well as vacuum filtered through 0.22 μm nylon membranes. The system was equilibrated with mobile phase MDTM (Thermo Scientific), through which mobile phase was pumped at a rate of 1.0 mL/min. with the oven held at 40⁰C and an analysis time of 10 mins. Eluting compounds were detected with a two-channel coulometric cell (6011RS, Thermo Scientific) and the cell potential was set at E1 = -175 mV (100 nA), E2 = +475 mV (1 µA). Data were acquired and processed with Chromeleon software version 7.2.8 (Thermo Scientific). Peaks from samples were then identified and quantified by comparison with the 5-HT standard (Sigma, St. Louis, MO).

***Immunofluorescence Assay***

Cells were grown in 16-well CultureWell™ Chambered Coverglass (Cat#: C37000, ThermoFisher) for all immunofluorescence (IF) assays. For IF staining, cells were fixed with 4% (w/v) methanol-free formaldehyde (Cat#: 28906, ThermoFisher) in 1 × PBS at room temperature for 15 mins, followed by 3 ice-cold PBS washes. Cells were processed with or without 0.1% Triton X-100 permeabilization. Fixed cells were blocked with 3% BSA in PBS for 30 minutes and were then incubated with primary antibodies overnight at 4°C. After removal of primary antibody and rinsing with PBS 3 times, cells were incubated with appropriate fluorochrome-conjugated secondary antibody for one hour at room temperature. The cells were stained with DAPI and mounted with Prolong® Gold Antifade Reagent (Cat#: 9071S, Cell Signaling) before visualization using a Zeiss LSM 780 Confocal Microscope. Antibody information, including vendor’s catalog numbers and dilutions are listed in **Supplementary Table S1b**.

To visualize the mCherry-tagged ERICH3 (mCh-ERICH3) and GFP-tagged CLTC (GFP-CLTC) in SK-N-SH cells, plasmids designed to overexpress mCh-ERICH3 and GFP-CLTC were co-transfected into cells as described above. The GFP-CHC17KDP cDNA plasmid for overexpressing GFP-CLTC fusion protein was a gift from Stephen Royle (Addgene plasmid # 59799)^9^. The mCh-ERICH3 and GFP-CLTC in cells were recorded using a Zeiss LSM 780 Confocal Microscope at one second interval for 200 seconds at 100× magnification.

***Association between ERICH3 SNP and Antidepressant Response***

The *ERICH3* rs11580409 SNP was tested for association with antidepressant treatment outcomes in four independent GWA studies: STAR*D^10^, ISPC^11^ PReDICT^12, 13^, and PGRN-AMPS^14, 15^. STAR*D used primarily the 16-item Quick Inventory of Depressive Symptomatology (QIDS-C16) to measure depression symptoms at baseline and follow-up visits after treatment with SSRIs. Details of the STAR*D study have been reported elsewhere^10, 16^. A total of 1578 subjects, who had genomic data as well as both baseline and 6-week QIDS-C16 measurements, were included in the association analysis. Analysis was adjusted for the first seven eigenvectors constructed from genome-wide SNP data because the STAR*D subjects included multiple ancestral backgrounds. The ISPC study recruited MDD patients from seven sites in the USA, Germany, and East Asia. All patients were studied using the 17-item Hamilton Depression Rating Scale (HAM-D) before and after SSRI treatment^11^. The association analysis was performed for 865 subjects who had genomic data as well as baseline and 4-week HAM-D scores. All analyses were adjusted for age, gender and sample site. The PGRN-AMPS recruited MDD patients from the Mayo Clinic Rochester, MN. All patients are European-Americans and were studied using the 17-item Hamilton Depression Rating Scale (HAM-D) before and after SSRI treatment^14, 15^. The association analysis was performed for 398 subjects who had genomic data as well as baseline and 8-week HAM-D scores. The PReDICT study (ClinicalTrials.gov Identifier: [NCT00360399](https://clinicaltrials.gov/ct2/show/NCT00360399?term=NCT00360399)) recruited treatment-naïve participants with primary diagnosis of nonpsychotic depression. All patients were given written, informed consent prior to beginning study procedures. Study approval was granted by the Emory Institutional Review Board and the Grady Hospital Research Oversight Committee. A detailed description of PReDICT study, including design, participants enrollment, treatment and outcomes, has been published previously^12, 13^. Genotyping, quality control and SNP imputation for DNA samples from the PReDICT study have been described elsewhere^17^. In the present study, a total of 151 patients who were treated with either escitalopram or duloxetine, and had genomic data as well as baseline and 12-week HAM-D scores were included in the analysis. Age, gender and treatment (either duloxetine or escitalopram) were controlled in the analysis. “Response” in all four studies was defined as ⩾50% reduction in depression scores after antidepressant treatment. Results of those four studies were combined for an inverse-variance weighted fixed-effects meta-analysis using the “rmeta” package in R. For each of the studies, response at the last-available time point after antidepressant treatment was included in the meta-analysis (STAR*D at 6 weeks, ISPC at 4 weeks, PReDICT at 12 weeks and PGRN-AMPS at 8 weeks).

**References:**

1. Darmanis S, Sloan SA, Zhang Y, Enge M, Caneda C, Shuer LM *et al.* A survey of human brain transcriptome diversity at the single cell level. *Proc Natl Acad Sci U S A* 2015; **112**(23)**:** 7285-7290.

2. Darmanis S, Sloan SA, Croote D, Mignardi M, Chernikova S, Samghababi P *et al.* Single-Cell RNA-Seq Analysis of Infiltrating Neoplastic Cells at the Migrating Front of Human Glioblastoma. *Cell Rep* 2017; **21**(5)**:** 1399-1410.

3. Dobin A, Davis CA, Schlesinger F, Drenkow J, Zaleski C, Jha S *et al.* STAR: ultrafast universal RNA-seq aligner. *Bioinformatics* 2013; **29**(1)**:** 15-21.

4. Anders S, Pyl PT, Huber W. HTSeq--a Python framework to work with high-throughput sequencing data. *Bioinformatics* 2015; **31**(2)**:** 166-169.

5. Song Y, Botvinnik OB, Lovci MT, Kakaradov B, Liu P, Xu JL *et al.* Single-Cell Alternative Splicing Analysis with Expedition Reveals Splicing Dynamics during Neuron Differentiation. *Mol Cell* 2017; **67**(1)**:** 148-161 e145.

6. Kharchenko PV, Silberstein L, Scadden DT. Bayesian approach to single-cell differential expression analysis. *Nat Methods* 2014; **11**(7)**:** 740-742.

7. Becht E, McInnes L, Healy J, Dutertre C-A, Kwok IWH, Ng LG *et al.* Dimensionality reduction for visualizing single-cell data using UMAP. *Nat Biotechnol* 2018; **37:** 38.

8. Harper S, Speicher DW. Purification of proteins fused to glutathione S-transferase. *Methods Mol Biol* 2011; **681:** 259-280.

9. Booth DG, Hood FE, Prior IA, Royle SJ. A TACC3/ch-TOG/clathrin complex stabilises kinetochore fibres by inter-microtubule bridging. *EMBO J* 2011; **30**(5)**:** 906-919.

10. Garriock HA, Kraft JB, Shyn SI, Peters EJ, Yokoyama JS, Jenkins GD *et al.* A genomewide association study of citalopram response in major depressive disorder. *Biol Psychiatry* 2010; **67**(2)**:** 133-138.

11. Biernacka JM, Sangkuhl K, Jenkins G, Whaley RM, Barman P, Batzler A *et al.* The International SSRI Pharmacogenomics Consortium (ISPC): a genome-wide association study of antidepressant treatment response. *Translational psychiatry* 2015; **5:** e553.

12. Dunlop BW, Binder EB, Cubells JF, Goodman MM, Kelley ME, Kinkead B *et al.* Predictors of remission in depression to individual and combined treatments (PReDICT): study protocol for a randomized controlled trial. *Trials* 2012; **13:** 106.

13. Dunlop BW, Kelley ME, Aponte-Rivera V, Mletzko-Crowe T, Kinkead B, Ritchie JC *et al.* Effects of Patient Preferences on Outcomes in the Predictors of Remission in Depression to Individual and Combined Treatments (PReDICT) Study. *The American journal of psychiatry* 2017; **174**(6)**:** 546-556.

14. Ji Y, Biernacka JM, Hebbring S, Chai Y, Jenkins GD, Batzler A *et al.* Pharmacogenomics of selective serotonin reuptake inhibitor treatment for major depressive disorder: genome-wide associations and functional genomics. *Pharmacogenomics J* 2013; **13**(5)**:** 456-463.

15. Mrazek DA, Biernacka JM, McAlpine DE, Benitez J, Karpyak VM, Williams MD *et al.* Treatment outcomes of depression: the pharmacogenomic research network antidepressant medication pharmacogenomic study. *J Clin Psychopharmacol* 2014; **34**(3)**:** 313-317.

16. Trivedi MH, Rush AJ, Wisniewski SR, Nierenberg AA, Warden D, Ritz L *et al.* Evaluation of outcomes with citalopram for depression using measurement-based care in STAR*D: implications for clinical practice. *The American journal of psychiatry* 2006; **163**(1)**:** 28-40.

17. O'Connell CP, Goldstein-Piekarski AN, Nemeroff CB, Schatzberg AF, Debattista C, Carrillo-Roa T *et al.* Antidepressant Outcomes Predicted by Genetic Variation in Corticotropin-Releasing Hormone Binding Protein. *The American journal of psychiatry* 2018; **175**(3)**:** 251-261.
